# Transcriptomic and proteomic analyses of seasonal photoperiodism in the pea aphid

**DOI:** 10.1186/1471-2164-10-456

**Published:** 2009-09-29

**Authors:** G Le Trionnaire, F Francis, S Jaubert-Possamai, J Bonhomme, E De Pauw, J-P Gauthier, E Haubruge, F Legeai, N Prunier-Leterme, J-C Simon, S Tanguy, D Tagu

**Affiliations:** 1 INRA, UMR 1099 BiO3P, F-35000 Rennes, France; 2 Gembloux Agricultural University, Department of Functional and Evolutionary Entomology, Passage des Déportés 2, B-5030 Gembloux, Belgium; 3 University of Liège, Mass Spectrometry Laboratory, Sart Tilman, Liège, Belgium

## Abstract

**Background:**

Aphid adaptation to harsh winter conditions is illustrated by an alternation of their reproductive mode. Aphids detect photoperiod shortening by sensing the length of the night and switch from viviparous parthenogenesis in spring and summer, to oviparous sexual reproduction in autumn. The photoperiodic signal is transduced from the head to the reproductive tract to change the fate of the future oocytes from mitotic diploid embryogenesis to haploid formation of gametes. This process takes place in three consecutive generations due to viviparous parthenogenesis. To understand the molecular basis of the switch in the reproductive mode, transcriptomic and proteomic approaches were used to detect significantly regulated transcripts and polypeptides in the heads of the pea aphid *Acyrthosiphon pisum*.

**Results:**

The transcriptomic profiles of the heads of the first generation were slightly affected by photoperiod shortening. This suggests that trans-generation signalling between the grand-mothers and the viviparous embryos they contain is not essential. By analogy, many of the genes and some of the proteins regulated in the heads of the second generation are implicated in visual functions, photoreception and cuticle structure. The modification of the cuticle could be accompanied by a down-regulation of the *N*-β-alanyldopamine pathway and desclerotization. In *Drosophila*, modification of the insulin pathway could cause a decrease of juvenile hormones in short-day reared aphids.

**Conclusion:**

This work led to the construction of hypotheses for photoperiodic regulation of the switch of the reproductive mode in aphids.

## Background

To adapt to hard winter conditions, many organisms living in temperate regions use photoperiod cues to anticipate the transition between autumn and winter. Such seasonal photoperiodism enables individuals to prepare winter installation through physiological or behavioural adaptations such as migration, hibernation or over-wintering egg-laying. Aphids are plant phloem feeding insects that provoke significant damage to agricultural crops. As poikiloterm animals, they do not regulate their internal temperature and die in cold winters. They bypass this difficulty by producing over-wintering eggs in the autumn that enter diapause during the winter period. Aphids are among the rare organisms practicing cyclical parthenogenesis during their annual life-cycle [1], alternating between viviparous parthenogenesis and oviparous sexual reproduction. In spring, eggs hatch and the new born aphids develop clonal colonies by parthenogenesis: viviparous females produce other viviparous females that are genetically identical, without haploid gamete formation or meiotic recombination [2]. At the end of the summer, these colonies produce, by clonal parthenogenesis, sexual morphs (males and oviparous females) that mate, these oviparous sexual females then lay eggs before winter.

In viviparous parthenogenetic aphids, embryos develop within the abdomen of their mother. Each mother contains several dozens of embryos at different stages of development. The most developed embryos have nearly complete differentiation of their ovaries with a germarium and several follicle chambers. Embryos at early stages are already formed within these follicle chambers. Thus, an adult viviparous female aphid contains two embedded generations: nearly fully developed embryos and early embryos within these developed embryos. This is the so-called "telescoping of generations".

The switch between parthenogenetic and sexual reproduction in aphids is driven by the variation of abiotic factors in autumn, primarily the photoperiod. Photoperiod shortening is sufficient to trigger the switch in the reproductive mode; decrease in temperature further promotes this switch [3]. Aphids measure the length of the night phase (scotophase); a minimum number of consecutive inductive nights is required to trigger the switch in the reproductive mode [4]. Several observations suggest that in aphids, part of the photoperiodic signal is detected by the protocerebrum in the brain through the cuticular head capsules [5,6]. Several aphid putative photoreceptors and transducer proteins have been located in the protocerebrum and the compound eyes in *Megoura viciae *[7]. Early transduction of the photoperiod signal involves a group of neurosecretory cells (Group I) located in the *pars intercerebralis *of the aphid protocerebum [8]. Transduction of the photoperiodic signal to the target tissues and cells located in the ovaries is still unresolved; however, ectopic applications of melatonin [9] or juvenile hormones [10,11] suggest that these molecules play key roles in the oocyte fate. During viviparous parthenogenesis, the photoperiodic signal may be detected and/or transduced through the different embedded generations; the regulatory mechanisms of such trans-generational signalling are not known.

Recently, with the development of genomic tools for the pea aphid *Acyrthosiphon pisum *[12,13], global analyses of gene regulation have been undertaken between aphids producing or not sexual. A receptor of GABA whose mRNA is up-regulated in long-night reared insects has already been identified [14]. Our group was the first to demonstrate that genes encoding cuticle and signalling proteins are regulated by shortening of the photoperiod [15,16]. To date, these studies have been performed on one development stage and during only one generation.

Herein, we analysed the transcriptomic and proteomic response of the pea aphid to shortening of the photoperiod at different stages covering the two parthenogenetic generations required before the birth of the future sexuals. We observed very few transcripts were regulated in the heads of the grand-mothers. In contrast, major changes occurred in the heads of mothers of the future sexual; these are probably linked to the developmental program of parthenogenetics that are sexual producers. Genes with putative functions in visual cues, photoreception, cuticle structure and the insulin pathway are particularly discussed.

## Results

Microarray and DIGE experiments were performed in order to identify gene and protein expression profiles accompanying the switch from asexual to sexual reproduction induced by a shortening of the photoperiod in aphids. All RNA and protein samples were collected from dissected pea aphid heads in order to focus on the early steps of the photoperiodic signal detection and transduction, and to eliminate RNAs and polypeptides of the next generation contained within the abdomen.

### Slight transcriptomic response in the heads of the grand-mother generation

**Figure 1:**
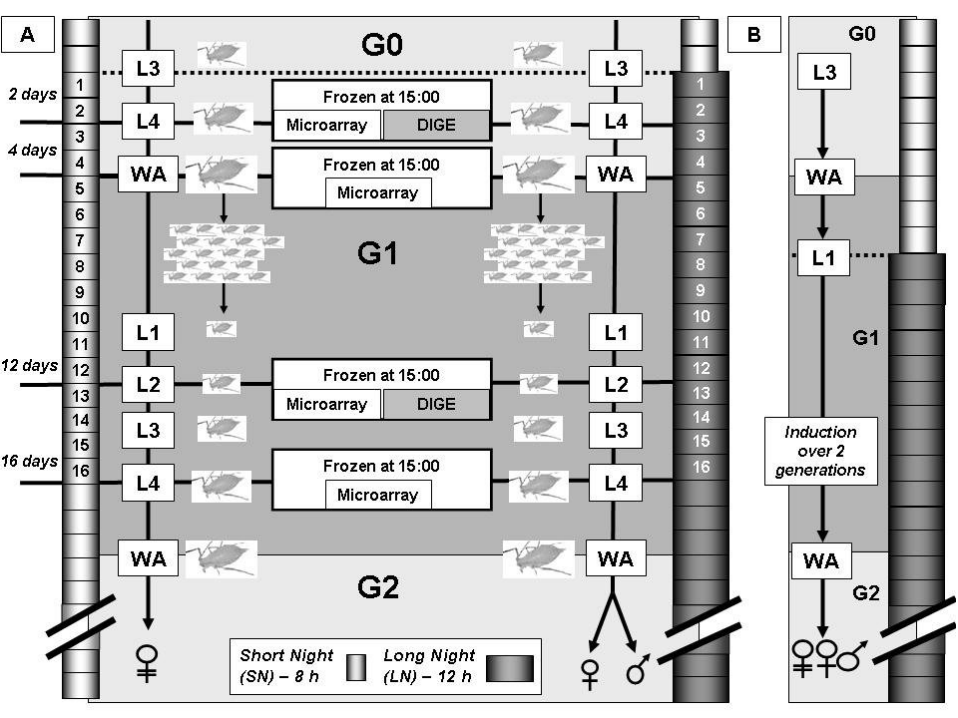
**Biological experiments performed to collect material for microarray and DIGE experiments**. **A**: L3-G0 aphids initially reared under Short Nights were separated into two batches, one reared under Long Nights (LN: 12 h of light, and 12 h of night) and the other remaining under SN conditions. For microarray experiments, when aphid reached L4-G0 and WA-G0 (Wingless Adult -- G0) stages, 25 aphids per batch were collected and immediately frozen. Once remaining, WA-G0 individuals began to lay their offspring; one L1-G1 was kept per adult (one L1 per plant) and stages L2-G1 and L4-G1 stages were collected (25 aphids per batch) and immediately frozen. Similar but independent experiments were performed to collect material for DIGE analyzes. Aphids were collected at only 2 stages (L4-G0 and L2-G1) for proteomic analyzes. **B**: as in A, except that the induction was initiated in L1-G1 generation.

**Figure 2:**
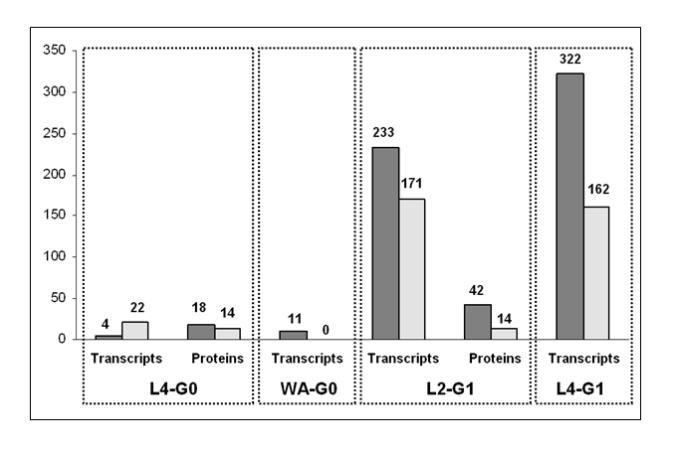
**Number of significantly regulated transcripts and proteins per each stage**. Numbers upon the bars indicate the number of regulated transcripts or proteins after statistical analyses (see methods). Dark areas: up-regulated transcripts or proteins; grey areas: down-regulated transcripts or proteins.

Transcript profiles between Long-Night (LN) or Short-Night (SN) reared aphids were compared at different stages of production of sexual individuals: two stages for generation G0 (L4 and wingless adult (WA) grand-mothers) and two for G1 (L2 and L4 mothers) (Figure 1). Of the 7166 spotted cDNAs, 6766 (94.4%) passed the quality filters of image analysis and normalization at L4-G0 stage, 6554 (91.5%) at WA-G0 stage, 6495 (90.6%) at L2-G1 stage and 5857 (81.7%) at L4-G1 stage. Statistical analyzes were performed on log-ratios of normalized values of fluorescence of LN RNA samples and of SN RNA samples. Four independent analyses (one per developmental stage) were performed using the SAM software, and the statistical test was of one-class response type (Figure 2). 26 transcripts were detected as regulated at L4-G0 stage (FDR = 2.5%), 11 transcripts (FDR = 6.5%) at WA-G0 stage with high FDR, indicating that a very low number of transcripts are regulated in G0. The selection of high FDR was necessary to obtain significantly regulated genes, since a lower FDR value provided no significantly regulated genes in the G0. In contrary, 404 transcripts (FDR = 0.1%) at L2-G1 stage and 484 (FDR = 0.1%) at L4-G1 with low FDR. These changes of expression revealed that the major transcriptomic modifications occurred in heads of the mothers of the future sexuals (G1), whereas few transcripts were differentially expressed in heads of the grand-mothers (G0) of the future sexuals (less than 0.4% of the spotted cDNAs with high FDR).

The DIGE analysis performed at L4-G0 and L2-G1 stages identified 58 significantly regulated polypeptides at the G1 generation, and only 32 at the G0 generation (Figure 2). This corresponds to 4.5% of the total proteins detected on a 2D-gel, whereas nearly 10% of the transcripts captured by the microarrays were regulated.

### Induction of sexual morphs accross 2 generations

Three generations (grand-mothers, mothers and the sexual individuals) were used to collect samples for microarray hybridizations and DIGE analyses. This protocol ensured that the complete chain leading to the production of sexual individuals was obtained (Table 1). The low level of regulated transcripts in the heads of the first generation supported Lees' hypothesis [17] that there was little or no grand-mother effect on the detection of the photoperiod changes. Consequently, under LN conditions, the embryos within the grand-mother might directly detect the photoperiod shortening through the abdominal cuticle of the grand-mother.

**Table 1:** Induction of sexual morphs across 2 generations.

**Induction**	**Individuals **	**Sexual producers**	**Parthenogenetic-producers**
2 generations (18°C)	25	96%	4%
2 generations (15°C)	65	98.5%	1.5%
3 generations (18°C)	15	100%	0%
3 generations (15°C)	15	100%	0%

The offspring of individuals reared under LN conditions at L1 stage (2 generations) at 2 distinct temperatures (15 and 18°C) was analyzed in terms of percentage of sexual (oviparous and male)-producers or parthenogenetic-producers. The offspring of control individuals maintained under continuous LN conditions across 3 generations is also indicated for comparison.

To experimentally eliminate the grand-maternal effect, new-born L1 were directly transferred from SN to LN conditions. Once they reached adulthood, their offspring was analyzed for production of sexual morphs. In this experiment, the induction process took place across only 2 generations (Figure 1B). Progeny analyses showed that at 18°C or 15°C (in order to slow down embryo development, and thus artificially expand the number of inductive LN experienced), 96% and 98.5% of the individuals respectively were already sexual-producers. In both cases, the most of the sexual individuals were males (data not-shown). These results suggest that even when the number of sexual females was decreased, production of sexual morphs was possible in two generations: this confirms the hypothesis that signalling from the grand-mother is not essential for the switch in the reproductive mode in the pea aphid.

### Functional annotation of regulated polypeptides

**Figure 3:**
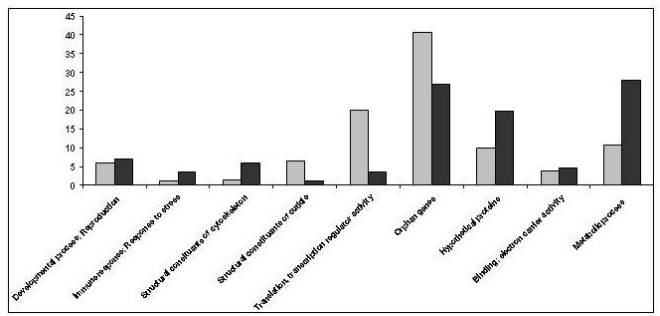
**Distribution into functional categories of the significantly regulated proteins and transcripts**. 9 categories have been selected using the GO terms. "Orphan gene" category corresponds to polypeptides sharing no homologies with known proteins whereas "Hypothetical" category contains polypeptides sharing homologies with protein of unknown function. GO0035502: Developmental process; GO0000003: Reproduction; GO0006955: Immune response; GO0006950: Response to stress; GO0005200: Structural constituants of cytoskeleton; GO0042302: Structural components of cuticle; GO0030528: Transcription regulatory activity; GO0045182 Translational regulatory activity; GO0005488: Binding; GO0009055: Electron carrier activity. GO0008152: Metabolic process. Y axis: percentage of regulated proteins or transcripts for each category. Grey areas: regulated transcripts; dark areas: regulated polypeptides.

A search for homologies in the NCBI non-redundant database and in a home-made pea aphid database was performed for the 86 regulated polypeptides. About 73% (63 polypeptides) of the proteins shared homologies with identified proteins, and 27% (23 polypeptides) corresponded to orphan genes (Additional File 1). Approximately 70% of these proteins were up-regulated by the photoperiod shortening. Fold changes of differentially expressed polypeptides ranged from 1.1 to 2.5 for those up-regulated and -4.5 to -1.1 for those down-regulated. Based on sequence homology, classification of polypeptides into functional groups (Figure 3) indicated that 28% of the regulated polypeptides were involved in "Metabolic Process". In addition to this strong molecular signature, 1.2% (1 protein) corresponded to "Structural Constituents of Cuticle", 3.5% to "Translation, Transcription Regulatory Activity", 3.5% to "Immune Response and Response to Stress", and 4.7% to "Binding and Electron Carrier Activity". 5.8% of identified polypeptides are implicated in "Structural Constituents of Cytoskeleton", and 7% in "Development Process and Reproduction". The "Metabolism" thus corresponds to the largest group, indicating a large modification of the metabolism in short-day reared insects.

### Functional annotation of regulated transcripts

A search for homologs in the non-redundant database was performed for the 616 different transcripts selected as undergoing significant regulatory changes. Functional annotation was assisted by data from Flybase and Uniprot databases, supported by the Gene Ontology classification. About 59.3% of the transcripts shared homology with identified genes, and 40.7% corresponded to orphan genes (Additional File 2). Approximately half of the transcripts were either up-or down-regulated by photoperiod shortening. Fold changes of differentially expressed transcripts ranged from -1.3 to -500 (mean: -5.5) for down-regulated genes and from +1.3 to +250 (mean: +2.7) for up-regulated genes. Classification of transcripts into different functional groups (Figure 3) indicated that 20% of the transcripts were involved in "Translation, Transcription Regulatory Activity" (including an important number of ribosomal proteins), 3.7% in "Binding and Electron Carrier Activity" and 10.7% in "Metabolic Process". 1.3% of the regulated transcripts were involved in "Structural Constituents of Cytoskeleton" and 1% in "Immune Response and Response to Stress". More strikingly, the analyses revealed that 6.5% of the regulated genes were involved in "Structural Constituents of Cuticle": 38 transcripts corresponded to cuticular proteins, 2 transcripts encoded enzymes involved in chitin metabolism and 3 were homologous to glycin rich proteins, known to be major constituents of the cuticle [18]. The majority of arthropods cuticular proteins contain a conserved region known as the Rebers and Riddiford consensus (RR consensus). Three distinct forms of this extended consensus have been defined [19]: RR1, RR2 and RR3. A search of such motifs using the cuticleDB website [20] indicated that of the 38 transcripts encoding cuticular proteins, 18 contained a RR2 domain, 7 contained a RR1 domain whereas 13 did not contain any of the RR domains (Additional File 3).

Apart from this strong biological signature corresponding to the differential expression of cuticular protein genes in our experiment, 6% of the differentially expressed transcripts corresponded to genes known to be involved in "Development and Signalling" (Table 2). Eight transcripts had homology with proteins involved in nervous system development, from axon guidance and crossing to synaptogenesis. Seven transcripts had homology with proteins implicated in the visual system (essentially eye development and phototransduction), 3 with proteins mediating neurotransmission, 3 with proteins involved in hormonal regulation and 1 regulated by the circadian rhythm.

**Table 2:** List of regulated transcripts and proteins sharing homologies with proteins involved in signalization and nervous or visual system.

**ID**	**Transcript (T) or Protein (P)**	**Gene/protein name**	**Drosophila transcript** **or Protein accession**	**Function**	**Stages and level of regulation**
***Nervous system***					
DY229249.1	T	Rho1	CG8416	Axon guidance	L4G1 (5.1)
CL117Contig1	T	Neural Lazarillo	CG33126	Axon guidance	L2G1 (17.1) - L4G1 (15.4)
CL922Contig1	T	Capulet	CG5061	Midline axon crossing	L4G1 (1.7)
CL903Contig1	T	Argonaute 1	CG6671	Neural development and synaptogenesis	VAG0 (1.25) - L2G1 (-1.4)
DY230264.1	T	Brain tumor protein	CG10719	Neural proliferation in larval central brain	L2G1 (-1.7)
CL33Contig1	T	Calreticulin	CG9429	Peripheral nervous system development	L2G1 (2.4) - L4G1 (2.5)
CL505Contig1	T	Hdd11	O96382	Nervous system development	L2G1 (-1.8) - L4G1 (-2.1)
CL1Contig1089	T	Wunen	CG8804	Cell signalling and axon guidance	L4G1 (4.7)
126330868	P	G protein-regulated inducer	XP_001375758	Neurite outgrowth	L2G1 (-1,2)
193613348	P	Rho GTPase-activating protein	XP_001950332	Neurite growth and axon guidance	L2G1 (-1,1)
***Visual system and photoreception***					
CN753062.1	T	Canoe	CG2534	Ommatidial rotation fly eye	L2G1 (1.4) - L4G1 (1.5)
CL3017Contig1	T	Black	CG7811	Visual signalization	L2G1 (-1.7) - L4G1 (-2.3)
CL4099Contig1	T	Arrestin 2	CG5962	Phototransduction of rhodopsin	L4G1 (1.4)
CL505Contig1	T	Inhibitor 2	CG10574	Inibitor of axon targeting in photoreceptor cells	L2G1 (5.8) - L4G1 (29.9)
CL1755Contig2	T	Ebony	CG3331	Transmitter secretion in photoreceptors	L4G1 (-2.1)
Ap_SDD3_6A12_SP6	T	cPka-1	CG4379	Eye development	L2G1 (1.4)
CL94Contig1	T	Calnexin	CG11958	Rhodopsin maturation	L2G1 (1.5) - L4G1 (6.5)
***Neurotransmission***					
CL5160Contig1	T	Dunc-13-4A	CG32381	Synaptic vesicle cycle	L2G1 (1.3) - L4G1 (1.3)
CL9681Contig1	T	Kinesin Dunc 10-4A	CG8566	Synaptic vesicle transport	L2G1 (-1.3) - L4G1 (-1.3)
CL6069Contig1	T	DEP containg domain	Q8CIG0	G-protein signalling and dopaminergic	L2G1 (-1.4) - L4G1 (-1.6)
CN752364.1	P	Kinesin Dunc 10-4A	Q8JIX1	Synaptic vesicle transport	L4G0 (1,2)
***Hormone and circadian rhythm***					
CL323Contig2	T	Insulin-degrading enzyme	CG5517	Degradation of insulin	L2G1 (1.3) - L4G1 (1.3)
DY230287.1	T	Insulin-like receptor	Q93105	Receptor of insulin	L2G1 (-1.5) - L4G1 (-1.6)
CL425Contig1	T	ImpL2	CG15009	Regulation of ecdyzone synthesis	L2G1 (-1.5) - L4G1 (-1.5)
CL1091Contig1	T	Dreg-5	CG2928	Circadian rhythm	L4G1 (2.7)

Regulated transcripts or proteins were divided into 4 categories: nervous system development, neurotransmission, visual system and others. The accession numbers of each contig, EST or GeneID and the accession number of the corresponding *D. melanogaster *transcripts (CG...) or protein (Q...) are indicated, as well as their putative function and their level of regulation at the corresponding stages.

Statistic analyses were performed to identify significant enrichment of gene families in the significantly regulated genes compared to all the genes spotted on the microarray. Each of the spotted genes with a homolog in *D. melanogaster *has been assigned the corresponding GO terms. From the initial set of 6776 spotted cDNAs and 619 regulated cDNAs, 2018 and 222 (respectively) were assigned at least one GO term. The list of significant terms is given in Additional File 4. This general analysis demonstrates enrichment in biosynthetic processes and confirms the enrichment in structural constituents of cuticle and ribosomes.

Despite the quantitative difference in the data, the combined transcriptomic and proteomic approach allowed the identification of common genes and proteins regulated by the photoperiod. This includes several heat shock or zinc finger proteins, translation initiation factors, protein kinases, myosin-like proteins and several other proteins from the general metabolism. Most interestingly, pea aphid homologs of kinesins putatively involved in synaptic vesicle transport, and Rho GTPases involved in axon guidance were regulated at both transcriptomic and proteomic level (see Table 2 and discussion).

## Discussion

The objective of our microarray and DIGE analyses was to identify cellular pathways regulated in the heads of the pea aphid during the switch from parthenogenesis to sexual reproduction. 5% of proteins and 10% of transcripts changed significantly in our experiments. The total protein extraction procedure does not allow an exhaustive extraction of the whole pea aphid proteome, and the microarray contained about 19% of the 34,000 predicted genes of the pea aphid genome. Thus, although this work allows high throughput analysis of protein and transcripts regulated during shortening of the photoperiod, it does not cover the whole proteome and transcriptome of the pea aphid. Nevertheless, the DIGE and transcriptomic analyses identified an important regulation of proteins involved in general metabolism and of transcripts corresponding to the protein synthesis machinery. This strong general signature indicates that the parthenogenetic morph producing sexuals that appear in the autumn has a different metabolism to the parthenogenetic morph producing parthenogenetic individuals, despite the absence of morphological differences between them.

### Not essential grand-mother effect

The process of induction of sexual morphs in our experimental conditions utilized three generations. The grand-mothers (G0) are the first individuals that experiments LN conditions. They are parthenogenetic and their embryos (G1) will be the parthenogenetic mothers of the future sexual individuals (G2). We thus analyzed DIGE and/or transcriptomic profiles of aphid heads at different developmental stages for each generation. The first conclusion from these analyses is that the transcriptome of the grand-mothers is only slightly modified by shortening of the photoperiod, suggesting, as hypothesized several years ago [17], that there is little or no transmission of the photoperiod signal from the grand-mother to the mother of the future sexuals. In aphids, a minimum number of consecutive LN is necessary to observe the irreversible production of sexual morphs. For the clone YR2 of the pea aphid and in our conditions, this number is 10 [15]. L4-G0 aphids were collected after 2 consecutive LN cycles and WA-G0 aphids after 4 cycles. It is thus possible that after such numbers of consecutive LN, the major modifications of gene regulation that lead to the production of sexual morphs are not initiated. Another possibility is that embryos of the future mothers could detect photoperiodic signals already within the abdomen of the grand-mothers, before their birth [17,21]. Indeed, we observed that an induction of sexual morphs across 2 generations (without the grand-maternal generation) was possible. The almost complete absence of transcriptomic and proteomic modifications in the heads of the grand-mothers suggests the embryos have the capacity to directly sense environmental cues through the cuticle of their mother before birth.

### Photoperiod shortening regulates the expression of transcripts involved in visual system and photoreception

**Figure 4:**
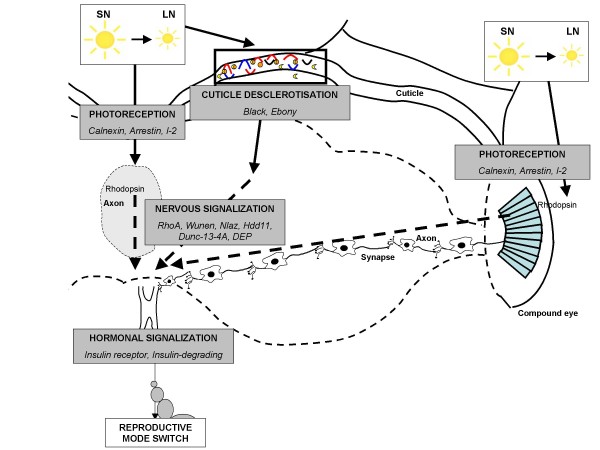
**Hypothetic model of seasonal photoperiodism transcriptomic regulation in the pea aphid's head**. This scheme corresponds to the head of the sexuparae submitted to photoperiod shortening and transmitting the signal to its embryos, the future sexuals. The photoperiod is sensed by still unknown photoreceptors located either in the brain (left side of the diagram) or in the compound eyes (right side). Perception is followed by a series of nervous signalling through different pathways such as Rho, Wunen or Dunc. In parallel, modification of the cuticle structure might lead to a higher concentration in dopamine in the brain that acts as a neurotransmitter. Nervous signalisation is relayed by endocrine regulation through the juvenile hormone signalling: the insulin pathway could be a regulator of the JH signalling pathway. SN: short night, LN: long night, cc: corpora cardiaca, ca: corpora allata.

Based on a similarity search, several of the significantly regulated genes corresponded to proteins known to be involved in photoreception or related to the visual system (Figure 4). Although aphid photoperiodic receptors are still uncharacterized, it is generally accepted that in Insects, rhodopsins are synthesized within neuron bodies of ommatidies and transported to their surface. *Calnexin*, which is up-regulated under LN conditions, is essential for rhodopsin maturation and transport [22]. Then, *arrestin2 *(also up-regulated) is translocated when rhodopsins are photoactivated by the light source [23]. This suggests an involvement of rhodopsin in the response of aphids to photoperiod shortening. Gao *et al*. [7] localized rhodopsin and arrestin polypeptides in both the compound eyes and the protocerebrum of the aphid *M. viciae*. Shiga and Numata [24] suggested that several photoreceptor systems could be involved in the phoroperiodic response in insects, implicating both intra-and extra-retinal components. In complement, the strongly up-regulated (*I-2*) is an inhibitor of *protein phosphatase-1 *(*PP1*) that is important for axon targeting of photoreceptor R-cells in *D. melanogaster *[25]. Finally, two transcripts related to the conjugation of β-alanine and dopamine shared similarity with the *black *and the *ebony *gene of *D. melanogaster*. *black *[26] and *ebony *mutants [27] respond abnormally to visual cues, suggesting a role in *D. melanogaster *visual system. These genes are also involved in cuticle structure (see below).

### Photoperiod shortening links cuticle modification and dopamine pathway

At least 38 pea aphid cuticular homologs were regulated by photoperiod shortening, some of these exhibit very high levels of differential expression (up to 500 times). Several genes have been already shown to be regulated by short days in the pea aphid; our data extends this observation to a larger number of cuticular protein genes [15,16]. The DIGE experiment did not show any regulation of cuticular proteins, probably because extraction of cuticular proteins requires specific procedures. Among the 38 cuticular proteins transcripts regulated by seasonal photoperiodism in this analysis, 25 contained a RR1 or RR2 domain.

The strong down-regulation of RR2 containing proteins suggests a modification of the cuticle. The cuticle is the storage site for several metabolites such as β-alanyldopamine (NBAD) [28]. NBAD forms electrostatic links between cuticular proteins and thus reinforces the cuticular matrix. If the down-regulation of cuticular proteins during photoperiod shortening is associated with a relaxation of the chitin-cuticular protein network, what is the fate of NBAD in such a cuticle? NBAD conjugation is driven by the *ebony *gene in *D. melanogaster*. In our experiment, we observed that the pea aphid transcripts homologs to *ebony *were down-regulated in L4-G1, suggesting that less NBAD is formed during photoperiod shortening. β-alanin is synthesized from aspartate under the control of *black *gene in *D. melanogaster*. The pea aphid transcripts homolog to *black *were down-regulated at L2-G1 and L4-G1 stages, suggesting that LN reared aphids synthesized less β-alanin. Consequently, a putative modification of the cuticle in response to day-length shortening could be related to a decrease of stored NBAD and β-alanin. This might result in the modification of dopamine concentration in aphid brains (Figure 4). This biogenic amine is a neurotransmitter and we suggest that it could play a role in the photoperiod signalling during the switch in the reproductive mode of the pea aphid.

### Photoperiod signal transduction involves the neuro-endocrine system

Many significantly regulated genes are putatively involved in nervous system development, supporting the hypotheses that nervous system structures are modified by seasonal photoperiodism [29]. A group of neurosecretory cells forming two clusters in the *pars intercerebralis *of the protocerebrum probably release neurosecretory material that could be transported along axon projections to targeted cells [8]. Several of the regulated genes showing homology to proteins implicated in axon guidance (e.g. *capulet, rho1*, *neural lazarillo*) [30-32] or the development of the central nervous system and synapses such as *wunen *or *HDD11 *[33-35] might be involved in this process. These two transcripts had already been detected as regulated at L3-G1 stage in earlier experiments [15], which supports their putative involvement in the transduction of the photoperiodic signal. DIGE experiments also detected the differential expression of 2 proteins, a G-protein-regulated inducer and a Rho-GTPase-activating protein, known to be involved in neurite growth in *Drosophila*, again supporting the role of the nervous structures in this mechanism.

Neurotransmitters might also be part of the transduction pathway of the photoperiodic signal. *dunc-13-4A *and *dunc 10-4A *are involved in synapse vesicle release [36] and *DEPcontaining protein *is probably involved in dopaminergic transmission [37]. Both microarray and DIGE experiments detected a differential expression of the transcript/protein homolog to Dunc 10-4A, indicating its possible importance in the response of the pea aphid to photoperiod shortening.

It has been previously suggested that viviparous parthenogenetic aphids reared under long nights and giving birth to sexual morphs have lower concentration of juvenile hormone (JH) than aphids reared under short nights [38]. In *D. melanogaster *mutations in insulin signalling pathway alter JH synthesis [39]. In our experiment, two genes related to proteins of the insulin pathway were detected as significantly regulated. One is a putative insulin receptor that is down regulated and the second a putative insulin degrading enzyme that is up-regulated. This suggests a link between insulin pathway and JH in seasonal photoperiodism of the pea aphid, as already shown in the mosquito *Culex pipiens *for diapause, or in the honey bee for cast determination [40,41].

## Conclusion

General transcriptomic and proteomic analyses strengthen the observation that aphid' embryos can detect seasonal photoperiodism directly within their mother and that signalling between grand-mothers and mothers is not essential. Several genes putatively involved in photoreception and neuro-endocrine signalisation have been identified. We propose a working hypothesis linking photoreception, cuticle modification and neuro-endocrine signalization in response to photoperiod shortening.

## Methods

### Biological experiments

All aphids were reared on *Vicia fabae *plant inside regulated cabinets. Biological material for microarray experiments was prepared under two daily photoperiodic regimes both at constant temperature of 18°C: i) "Short Night" (SN) at 16 h of light and ii) "Long Night" (LN) at 12 h of light to induce the production of sexual morphs. The overall experiment is described in the flow diagram of Figure 1. To initiate the experiment, two groups of 105 L3 larvae were placed either under SN or LN condition. This corresponds to generation G0 (Figure 1A). At the middle of the photophase, 25 individual were frozen when they had reached both the L4 and the wingless adult (WA) stages, in the two photoperiod conditions. The 55 remaining WA individuals (still divided in two groups) were left on 55 plants to lay their offspring: one larva of the 1st stage (L1) was kept per WA. This larva was selected among the 20 first born larvae. This is the generation G1 (Figure 1A). At the middle of the photophase, 25 individual were frozen when they had reached both the L2 and the L4 stages, in the two photoperiodic conditions. Thus, 25 individuals from 4 different stages (L4G0, WA-G0, L2-G1 and L4-G1) were collected in the two photoperiod conditions, forming the 8 samples used for microarray experiments. Five individuals per photoperiod condition were left on plants to reach adulthood: their progeny were analyzed to confirm the production of parthenogenetic individuals under SN condition and sexual morphs (males and oviparous females) under LN condition. The head of all frozen individuals were cut on a liquid nitrogen layer and used as starting material. This biological experiment was performed in triplicate.

Biological material for proteomic analyzes was harvested following the same protocol in independent experiments, except that only two stages were analysed: L4-G0 and L2-G1.

### Induction of sexual morphs across 2 generations

In the previous experiments, the induction protocol of sexual morphs (LN conditions) was applied for 3 generations. To test the possibility that sexual morphs could be reduced by a protocol applied for 2 generations, 25 WA (1 per plant) reared under SN conditions at 18°C were followed day-by-day and their first L1 (G1 generation) offspring was isolated and directly transferred to LN conditions at 18°C (Figure 1B). Once they reached adulthood, the progeny of these 25 individuals was analyzed in terms of production of sexual (oviparous females and males) or asexual morphs. The same experiment was also performed at a temperature of 15°C and the offspring of 65 individuals was analyzed.

### Microarray experiments cDNA microarray construction

A cDNA microarray was constructed from 7166 cDNAs and 49 controls, spotted in duplicate for a total of 14,430 spots. The array is described also at GEO (GPL8426). 6650 cDNAs were selected after EST clustering from cDNA libraries of antennae, digestive tract, head and salivary glands of the pea aphid [42]. A small number (126) of cDNAs corresponded to sequences obtained after differential display or subtractive hybridization experiments between SN and LN reared pea aphids [14,16]. These 6776 pea aphid cDNAs correspond to different transcripts of the pea aphid and migh represent approximately 19% of the predicted genes for the pea aphid genome. The 390 remaining cDNAs were selected from a cDNA library of the green peach aphid *Myzus persicae *[[43] and Karl Gordon, personal communication CSIRO]. The 49 controls consisted of 16 spots of fluorescent dyes (Cyanine 3), 3 buffers used for cDNA resuspension, 3 poly-A, 3 poly-T, 3 poly-linkers for the plasmid pDNR-lib (Clontech), 3 poly-linkers for the plasmid pTriplEX-2 (Clontech), and 18 *Arabidopsis thaliana *spike controls from the SpotReport-3 Array Validation System (Stratagene, CA, USA). cDNA probes were printed on Corning UltraGAPS II slides (Corning, NY, USA) with a Spotter Microgrid II (*Bio*robotics, Cambridge, UK) available at the Biogenouest transcriptomic facilites (UMR 6061, University of Rennes).

### RNA extraction, amplification and labelling

RNAs were extracted from heads of collected aphids using the SV Total RNA Isolation kit (Promega, Madison, WI, USA). The integrity and quantity of RNAs were verified using a Bioanalyser 2100 (Agilent Tech. Inc., Palo Alto, CA, USA). All RNAs collected at the four developmental stages were amplified using the MessageAmp aRNA kit (Ambion, Austin, Texas, USA), starting with 1 g of total RNA. Amplified RNAs (aRNAs) were quantified with a Nanodrop (Agilent). aRNAs (1.5 g) were labelled and purified with the ChipShot Indirect Labelling and Clean-Up System (Promega, Madison, WI, USA). Labelling was performed with the CyDye (Cy3/Cy5) Reactive (Amersham Biosciences-GE, Faifield, CT, USA).

### Microarray hybridizations

Hybridizations were performed with a Discovery XT System Hybridization Robot using the ChipMap 80 kit (Ventana Medical Systems, Tucson, AZ, USA) at INRA-SCRIBE transcriptomic facilities (IFR 140 GFAS, Rennes). Prehybridization was performed at 42°C for 1 h in a 0.5% BSA, 2× SSC and 0.2% SDS prehybridization buffer. Target labelled cDNAs were mixed before hybridizations at 42°C for 6 h (protocol no. 2, ALC-D60/10-H48/8, Ventana) in a ChypHybe80 (Ventana Medical Systems, Tucson, AZ, USA) hybridization buffer. Hybridized slides were washed manually with a RiboWash solution (2 times) and a 0.1× SSC solution (1 time). The 3 washes were performed at room temperature for 2 minutes.

Hybridizations were performed between samples extracted at LN and SN for each developmental stage (L4-G0, WA-G0, L2-G1 and L4-G1). No cross-hybridizations between stages were performed. For instance, the LN sample extracted from the L4-G0 stage was directly hybridized against the SN sample extracted from the L4-G0 stage. Combining these 4 stages with the three biological replicates and the dye swap produced a total of 24 slides.

### Data deposition

All the microarray data and procedures were deposited in the Gene Expression Omnibus database under the accession numbers GPL8426 (platform), GSM390951-GSM390974 and GSM390979 (samples), and GSE15776 (series).

### Data analysis

All fluorescent images of the microarrays were generated by a GenePix 4000B scanner and treated by the Genepix Pro analysis software v6.0 (Axon Instruments, Molecular Devices Co., Sunnyvale, CA, USA). Raw data were corrected by the MADSCAN software [44] as described [15]. Briefly, fluorescence background was subtracted and a spatial normalization was used before a scaling of the variance within each slide and between all the groups of 2 "dye-swaped" slides. After detection of the outliers, the normalized values of log-transformed ratios of fluorescence of LN samples on SN samples were used to perform a statistical analysis with SAM (Significance Analysis of Microarray) [45]. The 4 stages were analyzed independently: for each stage a "one class" response type analysis was performed. To detect genes significantly regulated, the same delta-value was applied for the 4 analyzes, which resulted in a maximum False Discovery Rate (FDR) of 6.5%. As some transcripts detected as significantly regulated by SAM analyses exhibited very low regulations factors (from 1.1 to -1.1), we only conserved genes as differentially expressed those with a factor of at least 1.3 or -1.3. Consequently, all selected transcripts exhibited a change of at least 30% in their expression level. Functional annotation was performed by blast searches. Pea aphid transcripts having similarities with *D. melanogaster *genes were annotated through FlyBase, whereas pea aphid transcripts with no *Drosophila *gene similarities were annotated at Uniprot. Each of the sequences was assign the corresponding GO numbers. Research for significant enrichment of GO terms in the regulated gene set was performed through the Babelomics platform [46]. For each EST spotted to the array, the corresponding predicted genes were search by mapping to the pea aphid genome at AphidBase [47]. The corresponding *D. melanogaster *homologs were retrieved for the PhylomeDB at AphidBase. The Flybase identifiers were loaded in Amigo [48] to retrieve the corresponding GO terms. The functional enrichment analysis was performed by comparing the two lists of genes; the spotted cDNAs and the significantly regulated cDNAs by means of a Fisher's exact test at a p-value < 0.05.

### Proteomic experiments

#### Analytical 2-D gel electrophoresis

Twenty five aphid heads per treatment were crushed in a 7 M urea, 2 M thiourea 20 mM Tris pH 8.5 buffer including 2% CHAPS, and centrifuged at 15000 g, 4°C for 15 min. Supernatants were collected and proteins were precipitated using the 2D Clean Up Kit according to the manufacturer's instructions (GE Healthcare). Quantification of the precipitated proteins was performed using the RCDC quantification kit from Biorad. The protein extracts (aliquot of 25 μg) were labelled with one of three Cydye (GE Healthcare) following standard DIGE protocol. Samples to be compared and labelled with either Cy3 or Cy5 were mixed together with a total head aphid internal standard protein mixture labelled with Cy2. This mix of labelled proteins was adjusted to a volume of 450 μl that was used to rehydrate 24 cm IPG strips (pH 3-10 NL from GE Healthcare) for 12 h at 20°C and constant voltage of 50 V. Isoelectric focusing (IEF) was carried out at 200 V for 200 Vh, 500 V for 500 Vh, 1000 V for 1000 Vh and 8000 V for 60000 Vh at 20°C and a maximum current setting of 50 μA/strip in an isoelectric focusing unit from BioRad. Following IEF, the IPG strips were equilibrated for 15 min in 375 mM Tris (pH 8.8) containing 6 M urea, 20% v/v glycerol, 2% w/v SDS, and 130 mM DTT and then for a further 15 min in the same buffer except that DTT was replaced with 135 mM iodoacetamide. The IPG strips were then sealed with 0.5% agarose in SDS running buffer at the top of gels (240 × 200 × 1 mm) polymerized from 12% w/v acrylamide and 0.1% N,N'-methylenebisacrylamide. The second-dimensional electrophoresis was performed at 20°C in Ettan Dalt-six electrophoresis unit (GE Healthcare) at 25 W/gel for 5 h. Gels were scanned with a Typhoon fluorescence imager (Amersham) at wavelengths corresponding to each cydye. Images were analysed with Samespot 2D software version 3.1 (nonlinear) according to the manufacturer's instructions.

Comparisons were performed between samples extracted at LN and SN for each developmental stage (L4-G0 and L2-G1). As for the transcriptomic analysis, no cross-comparisons between stages were performed. Combining these 2 stages with the three biological replicates and 3 technical replications produced a total of 18 2D-gels.

### Protein identification

A preparative gel using a non-labelled 500 μg sample of aphid protein mixture was run according to the conditions above; proteins were stained by a conventional Coomassie Blue Colloidal. The protein spots of interest (i.e. differentially regulated) were manually excised from the gel. Excised gel plugs were washed 3 times with water and cysteins were reduced with a 10 mM DTT solution for 45 minutes at 56°C followed by alkylation with 50 mM iodoacetamide at room temperature in the dark. Digestion was performed overnight with 12.5 ng/μL of trypsin (Roche) in 100 mM ammonium carbonate buffer, pH 8.4. The resulting peptides were extracted with 1% formic acid in 5% acetonitrile. One microliter of each digestion solution was load on a pre-spotted Maldi plate (Bruker).

Peptide mass analysis was performed on a Bruker Ultraflex II TOF/TOF system. Mass data acquisition was performed in the mass range of 50 to 1700 m/z using the Standard-Enhanced mode (8,100 m/z per sec). For each mass scan, a data-dependant scheme picked the 3 most intense doubly or triply charged ions to be selectively isolated and fragmented in the trap and the resulting fragments were mass analysed using the Ultra Scan mode (50-3000 m/z at 26,000 m/z per sec).

Raw data were analysed and formatted (Data Analysis software, Bruker) for protein identification using the NCBI non-redundant protein database and the MS search algorithm on the Mascot search engine [48]. The 160,000 pea aphid available ESTs (Genbank, June 2008) were used to form contigs [42] and to construct a database that was used for peptide annotation by the MS search algorithm on the Mascot research engine [49]. The mass tolerance of sequence ions were set at 0.5 Da, and carbamidomethylation of cysteines and methionine oxidation were set as fixed and variable modifications, respectively.

## Authors' contributions

GLT performed most of the experimental work for transcriptomic data and analysed all the data. He wrote part of the manuscript and designed all the figures and tables. FF performed the DIGE experiments and analyzed them together with EH. They both took part on the writing of the manuscript. SJP was involved in transcriptomic analyses and writing of the manuscript. JPG and FL are bioanalysts and bioinformaticians involved in data analyses. EDP performed mass spectroscopy experiments. NPL, JB and ST are technicians who took part at different stages of the program (insect rearing, photoperiod experiments, molecular biology). JCS discussed the data. DT was in charge of the program and wrote the manuscript. All authors read and approved the final version of the manuscript.

## Supplementary Material

Additional file 1 **List of the 86 proteins regulated during the kinetics experiment.** Click here for file

Additional file 2 **List of the 616 transcripts regulated during the kinetics experiment.** Click here for file

Additional file 3 **Type of RR domain contained in the 38 regulated transcripts encoding cuticular proteins.** Click here for file

Additional file 4 **List of significant GO terms.** Click here for file
