# Supplementary material for: Transcriptomic and proteomic analyses of seasonal photoperiodism in the pea aphid
Source: BMC Genomics. 2009 Sep 29;10:456. doi: 10.1186/1471-2164-10-456 (PMC2763885; doi:10.1186/1471-2164-10-456)
Supplement: Additional file 1 — List of the 86 proteins regulated during the kinetics experiment. [file 1471-2164-10-456-S1.DOC]

**Additional File 1: List of the 86 proteins regulated during the kinetics experiment.**

| **GeneID or pea aphid Contig/EST name** | **Protein accession** | **Gene Name** | **L4-G0** | **L2-G1** |
| --- | --- | --- | --- | --- |
| **General metabolism** |  |  |  |  |
| CL1Contig181 | Q7Z161 | Ferritin |  | 1,1 |
| CL1Contig342 | Q9VRP2 | Peptidase |  | 1,2 |
| CL6473Contig1 | Q8IH17 | NAD+ synthase |  | 1,3 |
| gi|156545112 | XP_001601897 | Cell cycle checkpoint kinase 2 |  | 1,2 |
| gi|157132436 | XP_001662562 | Cytochrome c oxidase |  | 1,2 |
| gi|157137371 | Q17F22 | Purine biosynthesis protein 6 | -1,1 |  |
| gi|157167990 | XP_001663037 | Aconitase |  | 1,2 |
| gi|170036251 | XP_001845978 | Fatty acyl-CoA reductase 2 |  | 2,1 |
| gi|33772540 | AAQ54669 | Carbamoylphosphate synthase |  | -2,2 |
| gi|52630967 | AAU84947 | Thioredoxin peroxidise |  | -3,8 |
| gi|66513183 | XP_396855 | Tyrosine phosphatase |  | 1,2 |
| gi|109130182 | XP_001087741 | Acyl-Coenzyme A thioesterase 2 | -1,1 |  |
| gi|115551756 | Q05KC0 | Luciferase | 1,1 |  |
| gi|126325805 | XP_001364584 | Transferrin |  | 1,3 |
| gi|126337073 | XP_001362336 | Protein kinase | 1,1 |  |
| gi|149598210 | XP_001510017 | Caspase-14 |  | -3,2 |
| gi|177784102|gb|FF338864.1|FF338864 | Q7Q6I5 | Diacylglycerol kinase activity |  | 1,2 |
| gi|24651125 | NP_524550 | ATP synthase-gamma chain | 1,5 | 1,5 |
| gi|25149255 | NP_741537 | Protein kinase | 1,2 |  |
| gi|46997918|gb|CN586195.1|CN586195 | XP_001949805 | Chemosensory receptor |  | 1,2 |
| gi|494991 | AAA69816 | Cytochrome P450 |  | 1,3 |
| gi|60729673 | JC8017 | Beta-galactoside alpha-2,6-sialyltransferase | 1,1 |  |
| gi|89574495 | ABD76378 | Diacetyl/L-xylulose reductase |  | 1,2 |
| giI157114403 | XP_001652254 | Glutamate semialdehyde dehydrogenase | -1,2 |  |
| CL1820Contig3 | Q16LL6 | Binding protein |  | -2,4 |
| CL432Contig1 | Q058U1 | Protein binding |  | -1,4 |
| gi|110760089 | XP_393718 | Hillarin |  | 1,2 |
| gi|149751255 | XP_001492313 | Binding regulatory factor | 1,2 |  |
| **Defence and stress response** |  |  |  |  |
| CL1Contig597 | Q2WG65 | Heat shock protein |  | 1,3 |
| gi|157011470 | ABV00894 | Heat Shock Protein 70 |  | -1,3 |
| gi|23193450 | AAN14525 | Heat shock cognate 70 |  | 1,2 |
| **Translation, transcription and replication** |  |  |  |  |
| gi|157123196 | XP_001660054 | Helicase |  | 1,3 |
| CL3866Contig1 | Q8CFW0 | Translation initiation factor |  | 2,1 |
| gi|68391596 | XP_696563 | Translation initiation factor 2C |  | 1,7 |
| **Cuticle structure and synthesis** |  |  |  |  |
| gi|112983226 | NP_001036952 | Glycine rich protein |  | 1,3 |
| **Cytoskeleton and vesicles** |  |  |  |  |
| gi|156542010 | XP_001599268 | Paramyosin |  | 1,3 |
| gi|383039909 | / | Tropomyosin 3 |  | -3,4 |
| gi|157131823 | XP_001655953 | Tropomyosin |  | 1,1 |
| gi|47517361|gb|CN752364.1|CN752364 | Q8JIX1 | Kinesin-like protein Kif1b alpha - Dunc 104A | 1,2 |  |
| gi|51979106 | AAU20322 | Tropomyosin | 1,1 |  |
| **Development and signalization** |  |  |  |  |
| gi|2529508 | AAB81178 | Developmental orphan receptor 2 |  | 1,2 |
| gi|126330868 | XP_001375758 | G protein-regulated inducer of neurite outgrowth |  | -1,2 |
| gi|193613348 | XP_001950332 | Rho GTPase-activating protein 17 |  | 1,1 |
| gi|50344729 | NP_001002038 | Annexin A6 |  | 1,6 |
| gi|156549218 | XP_001601256 | Annexin IX-A |  | -1,3 |
| gi|20129193 | NP_608754 | Zinc finger protein | 1,2 |  |
| **Orphan genes or transcripts homologous to hypothetical proteins** | | | | |
| CL2392Contig1 | Q9W4F7 | Hypothetical protein |  | -4,5 |
| CL3665Contig1 | Q17QT0 | Hypothetical protein |  | 1,2 |
| CL5006Contig1 | Q4RJJ9 | Hypothetical protein |  | 1,5 |
| gi|110757717 | XP_001121524 | Hypothetical protein | 1,2 |  |
| gi|110769648 | XP_001123143 | Hypothetical protein |  | 1,1 |
| gi|126343503 | / | Hypothetical protein |  | 1,3 |
| gi|47087457 | NP_998627 | Hypothetical protein |  | 1,2 |
| gi|125823261 | XP_691768 | Hypothetical protein | -1,2 |  |
| gi|125987828 | Q5HYJ3 | Hypothetical protein | -1,6 | 1,7 |
| gi|156540664 | XP_001602696 | Hypothetical protein |  | -1,6 |
| gi|157752868 | XP_001680146 | Hypothetical protein | -1,2 |  |
| gi|177759790|gb|FF317184.1|FF317184 | Q9VRR3 | Hypothetical protein | 1,2 |  |
| gi|20129515 | NP_609701 | Hypothetical protein | 1,1 |  |
| gi|24652729 | NP_610680 | Hypothetical protein |  | 2,5 |
| gi|55815245|gb|CV849562.1|CV849562 | CAM36311 | Hypothetical protein | -2,5 |  |
| giI115532526 | NP_001040778 | Hypothetical protein |  | -4,5 |
| gi|89473740 | ABD72682 | Hypothetical protein | -1,2 |  |
| CL10830Contig1 | / | No hit | -1,3 |  |
| CL3826Contig1 | / | No hit |  | 1,3 |
| CL7770Contig1 | / | No hit |  | -1,7 |
| CL8485Contig1 | / | No hit |  | 1,2 |
| gi|109194860|gb|EC388476.1|EC388476 | / | No hit | -1,5 |  |
| gi|109195744|gb|EC388964.1|EC388964 | / | No hit | 1,1 |  |
| gi|109197225|gb|EC389765.1|EC389765 | / | No hit |  | 1,7 |
| gi|111157595|gb|EE261278.1|EE261278 | / | No hit |  | 1,3 |
| gi|111157708|gb|EE261391.1|EE261391 | / | No hit | 1,1 |  |
| gi|111159688|gb|EE263366.1|EE263366 | / | No hit |  | -1,6 |
| gi|112432965|gb|EE571311.1|EE571311 | / | No hit | -1,2 |  |
| gi|158212830|gb|EX615699.1|EX615699 | / | No hit | 1,2 |  |
| gi|158230828|gb|EX633697.1|EX633697 | / | No hit | 1,1 |  |
| gi|158241868|gb|EX644737.1|EX644737 | / | No hit | -1,2 |  |
| gi|177739488|gb|FF301882.1|FF301882 | / | No hit | -1,2 |  |
| gi|51973178|gb|CN254702.1|CN254702 | / | No hit |  | 2,5 |
| gi|51973345|gb|CN254869.1|CN254869 | / | No hit | -1,6 |  |
| gi|55811647|gb|CV845964.1|CV845964 | / | No hit |  | 1,2 |
| gi|83663005|gb|DW013413.1|DW013413 | / | No hit |  | 1,3 |
| gi|83663549|gb|DW013957.1|DW013957 | / | No hit |  | 1,3 |
| gi|83663675|gb|DW014083.1|DW014083 | / | No hit | 1,1 |  |
| gi|84647557|gb|DW361886.1|DW361886 | / | No hit | 1,1 |  |
| gi|84647684|gb|DW362013.1|DW362013 | / | No hit |  | 1,2 |

The GeneID of the corresponding proteins identified in the NCBI non-redundant protein database or the EST or contig name of the corresponding proteins identified through the pea aphid database, the protein accession number, the putative function of the protein, as well as the regulation factors at L4-G0 and L2-G1 stages are indicated.
