# Supplementary material for: Transcriptomic and proteomic analyses of seasonal photoperiodism in the pea aphid
Source: BMC Genomics. 2009 Sep 29;10:456. doi: 10.1186/1471-2164-10-456 (PMC2763885; doi:10.1186/1471-2164-10-456)
Supplement: Additional file 2 — List of the 616 transcripts regulated during the kinetics experiment. [file 1471-2164-10-456-S2.DOC]

**Additional File 2 - List of the 616 transcripts regulated during the kinetics experiment.**

| **General metabolism** | | | | | | | | | | | |
| --- | --- | --- | --- | --- | --- | --- | --- | --- | --- | --- | --- |
| **ESTs** | **Contig** | | **Uniprot Accession** | | **E-value** | | **Gene Name** | **L4-G0** | **A-G0** | **L2-G1** | **L4-G1** |
| ID0AAK4YA01CM1 | gi|86462529|gb|DY228401.1|DY228401 | | Q70ME8 | | 3.00E-07 | | Acid phosphatase 1 |  |  |  | 1.3 |
| ID0AAK1YM22CM1 | CL1Contig930 | | Q9VLS4 | | 2.00E-19 | | Acyl-CoA binding |  |  | 1.3 |  |
| ID0AAK4YG14CM1 | CL1Contig171 | | Q9VLS4 | | 6.00E-23 | | Acyl-CoA binding |  |  | 1.5 | 1.4 |
| ApHL3SD-XXIX-B1 | CL1Contig69 | | Q9VA94 | | 2.00E-89 | | Acyl-CoA desaturase |  |  | 1.3 | 1.4 |
| ID0AAK4YG19CM1 | CL116Contig1 | | Q66J33 | | 2.00E-52 | | Adenylate kinase |  |  |  | 1.9 |
| ID0AAK2YO01CM1 | CL169Contig5 | | Q16MV5 | | 1.00E-23 | | Aldehyde dehydrogenase |  |  |  | -1.6 |
| ApAL3SD-XX-E12 | CL1795Contig1 | | Q7KMM4 | | 1.00E-135 | | Alpha-glucosidase |  |  |  | 1.9 |
| ID0AAK6YL04CM1 | CL1Contig37 | | Q9VL27 | | 6.00E-56 | | Alpha-N-acetylgalactosaminidase |  |  | -1.6 | -1.9 |
| ID0AAK9YL14CM1 | CL407Contig1 | | Q7Q0U7 | | 0 | | ATP citrate lyase |  |  |  | 1.3 |
| ApHL3SD-XIX-C12 | CL1755Contig2 | | A7VLR0 | | 2.00E-48 | | Ebony |  |  |  | -2.1 |
| ID0AAK1YP09CM1 | gi|86461844|gb|DY227716.1|DY227716 | | P29126 | | 1.00E-11 | | Beta-xylanase |  | 3.4 | -47.5 | -10.3 |
| Ap_SDD2_3B05_T7 | No Contig | | P05990 | | 3.00E-20 | | Rudimentary |  |  | 2.1 |  |
| ApHL3LD-XI-E4 | CL673Contig2 | | Q9VXC4 | | 1.00E-105 | | Carboxypeptidase |  |  |  | 1.5 |
| ID0AAK1YF08CM1 | CL225Contig1 | | A8B9G3 | | 1.00E-154 | | Cathepsin |  |  | -2.6 | -4.3 |
| MpW-XVIII-F07 | CTG_MP_47.3-34966411 | | Q9W227 | | 5.00E-66 | | Peptidyl-prolyl cis-trans isomerase |  |  | -1.9 |  |
| MpW-XVII-C03 | CTG_MP_354.3-34600178 | | Q9W227 | | 9.00E-73 | | Peptidyl-prolyl cis-trans isomerase |  |  | 1.3 |  |
| ID0AAK6YI07CM1 | CL1895Contig1 | | A4V302 | | 1.00E-38 | | Dihydrofolate reductase |  |  |  | 1.5 |
| ID0AAK4YI14CM1 | CL1338Contig1 | | Q8IN02 | | 8.00E-65 | | Diphtine synthase |  |  |  | -1.3 |
| ID0AAK3YL17CM1 | CL10Contig2 | | Q9TWZ1 | | 1.00E-165 | | Disulfide isomerase |  |  | 2.4 | 2.3 |
| MpW-V-E05 | CL_MP_6527.3-34966061 | | Q95T75 | | 3.00E-08 | | Disulfide isomerase |  |  | 1.6 | 2.0 |
| ID0AAK5YG17CM1 | CL919Contig1 | | Q1HQY4 | | 1.00E-140 | | Glycotransferase |  |  | -2.1 |  |
| ID0AAK1YJ11CM1 | CL1608Contig1 | | Q567K2 | | 6.00E-73 | | Esterase |  |  |  | -1.8 |
| ApDT-XVIII-A11 | CL1016Contig1 | | Q503G0 | | 2.00E-53 | | Exonuclease |  |  |  | 1.4 |
| ID0AAK9YP04CM1 | gi|86464555|gb|DY230427.1|DY230427 | | Q9VHX7 | | 2.00E-78 | | Fatty acid elongase |  |  | -1.3 |  |
| ID0AFF15AA09CM1 | CL249Contig1 | | Q7QLH1 | | 1.00E-136 | | Glucose-6-phosphate 1-dehydrogenase |  |  | -1.6 | -1.6 |
| ApAL3SD-II-B11 | CL3017Contig1 | | Q24062 | | 2.00E-84 | | Glutamate décarboxylase |  |  | -1.7 | -2.3 |
| ApHL3LD-IX-D5 | CL846Contig1 | | Q9VWT1 | | 1.00E-161 | | Histidyl-tRNA synthetase |  |  |  | 1.9 |
| ApHL3SD-XIII-E3 | CL1035Contig2 | | Q9VB64 | | 1.00E-38 | | Hydrolase |  |  |  | 1.5 |
| ID0AAK8YK18CM1 | CL519Contig1 | | Q9VWF0 | | 5.00E-27 | | Hydrolase |  |  | -1.5 | -1.2 |
| ID0AFF7BB11CM1 | CL149Contig3 | | Q9VDC2 | | 7.00E-62 | | isopentenyl-diphosphate delta-isomerase |  |  |  | -1.4 |
| ID0AAK10YJ14CM1 | CL914Contig1 | | Q9VX25 | | 4.00E-73 | | Ligase |  |  |  | 3.4 |
| ApHL3SD-XXVII-F7 | CL4132Contig1 | | Q16IV9 | | 1.00E-111 | | Lin-19-like |  |  |  | 2.9 |
| ID0AAK1YF24CM1 | CL1Contig245 | | Q9V4E0 | | 0 | | NADH deshydrogenase |  |  | -2.0 |  |
| ID0AFF13BA07CM1 | CL451Contig1 | | Q17PM3 | | 1.00E-121 | | Nuclease |  |  | 1.4 | 1.8 |
| ID0AAK10YO21CM1 | CL1Contig1068 | | Q95RE4 | | 9.00E-38 | | Nucleic acid binding |  |  |  | 1.2 |
| MpW-IV-D06 | CL_MP_7229.3-34966320 | | O61231 | |  | | Nucleic acid binding |  |  | 1.3 |  |
| ID0AAK10YF04CM1 | CL237Contig1 | | A1Z6X0 | | 2.00E-82 | | Oxidoreductase |  |  | 14.7 |  |
| ID0AAK2YK24CM1 | CL1Contig411 | | Q9VES6 | | 2.00E-35 | | Oxidoreductase |  |  | -29.0 | -5.7 |
| ApHL3SD-V-C7 | CL166Contig1 | | Q8MS59 | | 5.00E-43 | | Oxidoreductase activity |  |  | -2.5 | -1.3 |
| ID0AAG10CE02CM1 | CL211Contig1 | | Q9W227 | | 5.00E-67 | | Peptidyl-prolyl cis-trans isomerase |  |  | 1.3 | 1.4 |
| ID0AAK7YB14CM1 | CL1Contig923 | | A8E774 | | 3.00E-74 | | Peptidyl-prolyl cis-trans isomerase |  |  |  | 1.3 |
| ID0AFF9DH07CM1 | CL1Contig31 | | Q4JI71 | | 7.00E-39 | | Peptidyl-prolyl cis-trans isomerase |  |  | 1.3 | 1.5 |
| ApAL3SD-I-C12 | CL669Contig1 | | O17452 | | 2.00E-75 | | Peritrophin |  |  | -1.6 | -2.7 |
| ApAL3SD-XII-H11 | CL3186Contig1 | | Q7QI30 | | 3.00E-61 | | Phosphocholine esterase (Paf-AH) |  |  |  | 1.6 |
| ID0AAK9YF23CM1 | CL147Contig1 | | Q7M451 | | 0 | | Phosphogluconate dehydrogenase |  |  | -1.4 | -1.6 |
| ID0AAK3YI11CM1 | CL528Contig1 | | Q9VW80 | | 4.00E-54 | | Phospholipase activity |  |  | 1.3 |  |
| ApAL3SD-XII-A9 | CL203Contig2 | | A4V1F9 | | 5.00E-10 | | PolyUbiquitin |  |  | -1.4 | -1.3 |
| ApHL3LD-XVII-F7 | CL3341Contig1 | | A1ZA47 | | 1.00E-96 | | Protein binding |  |  |  | 1.5 |
| ApHL3SD-XXXIII-E2 | CL19Contig2 | | Q9VAY2 | | 0 | | Protein folding |  |  | 1.3 | 1.3 |
| ApHL3LD-XI-F6 | CL1824Contig1 | | Q7QJS7 | | 4.00E-32 | | Protein metabolism |  |  | 1.3 |  |
| ID0AAK7YJ22CM1 | CL385Contig1 | | A3EXQ8 | | 6.00E-48 | | Putative serine/threonine kinase |  |  |  | 1.3 |
| ID0AFF9DD04CM1 | CL1398Contig1 | | Q9VEJ3 | | 5.00E-74 | | Pyrroline-5-carboxylate reductase |  |  | -1.1 | -1.4 |
| ID0AFF2BD05CM1 | CL3411Contig1 | | Q9VTY1 | | 8.00E-93 | | Retinal binding |  |  | 1.4 | -1.5 |
| ID0AAK2YA11CM1 | CL45Contig2 | | A4UZW2 | | 1.00E-173 | | S-adenosylmethionine synthetase |  |  |  | 1.3 |
| ID0AAK10YD24CM1 | CL35Contig3 | | A1ZEB9 | | 1E-13 | | Serpin |  |  |  | 1.5 |
| ID0AAK2YJ06CM1 | CL1Contig756 | | Q9VHA1 | | 7.00E-92 | | Spermidine synthase |  |  |  | 1.6 |
| ID0AAG11AA02CM1 | gi|82572063|gb|DV743719.1|DV743719 | | Q9VZF8 | | 4.00E-56 | | Sphingomyelin phosphodiesterase |  |  | 1.3 | 1.4 |
| ID0AAK9YH11CM1 | CL1Contig799 | | A4V391 | | 1.00E-113 | | Tcp1-like | -1.4 |  | -1.7 | -2.9 |
| ID0AAG11CF02CM1 | CL1Contig1042 | | A4V0H9 | | 8.00E-20 | | Thioredoxin 3 |  |  |  | 1.3 |
| ID0AAG10CC12CM1 | CL1Contig346 | | Q5XUA0 | | 1.00E-107 | | Thioredoxin peroxidase |  |  | 1.3 |  |
| ApHL3SD-VIII-A2 | CL518Contig1 | | Q8T4E1 | | 1.00E-122 | | Transamidase activity |  |  | -1.5 |  |
| ID0AAK2YD01CM1 | CL44Contig1 | | Q6ITV1 | | 5.00E-39 | | Transformer 2 protein |  |  |  | 1.3 |
| ApDT-XXXIV-F9 | CL1470Contig1 | | Q17N86 | | 9.00E-67 | | Ubiquitin-activating enzyme |  |  |  | 1.9 |
| ApAL3SD-XVIII-G10 | CL408Contig2 | | Q8SYG3 | | 3.00E-86 | | Ubiquitin-protein ligase |  |  |  | -1.3 |
| ID0AAK1YJ12CM1 | CL1Contig243 | | A4KYS3 | | 0 | | Vacuolar ATP synthase subunit C | -2.2 |  | -6.5 | -24.2 |
| ID0AAK7YK10CM1 | CL509Contig1 | | Q8I099 | | 3.00E-40 | | Vitamin E binding |  |  | -1.4 |  |
|  | | | | | | | | | | | |
| **Molecules and ions transport** | | | | | | | | | | | |
| **ESTs** | **Contig** | | **Uniprot Accession** | | **E-value** | | **Gene Name** | **L4-G0** | **A-G0** | **L2-G1** | **L4-G1** |
|  |  | |  | |  | |  |  |  |  |  |
| ApHL3LD-XI-H5 | CL1Contig589 | | Q699N4 | | 1.00E-57 | | Cytochrome oxidase subunit III |  |  |  | 2.6 |
| MpW-XIV-E05 | CL_MP_9054.3-34966562 | | B0XHS8 | | 9.00E-08 | | Cytochrome P450 |  |  |  | -1.6 |
| ID0AAK6YG21CM1 | CL274Contig2 | | Q6XIK5 | | 4.00E-69 | | Tango7 |  |  |  | 1.3 |
| ApAL3SD-VI-E3 | CL5030Contig1 | | Q9VKU1 | | 2E-13 | | Fatty acid transport protein |  |  | -1.3 | -1.3 |
| ApHL3SD-III-E2 | CL4439Contig1 | | A8JNH8 | | 2E-16 | | Glucose transporter |  |  |  | 1.3 |
| ID0AAK2YL04CM1 | CL5311Contig1 | | Q7QE81 | | 2E-15 | | Mitochondrial import inner membrane |  |  |  | 2.4 |
| ApHL3SD-XXXI-G9 | CL2329Contig1 | | Q5TZH9 | | 9E-14 | | Nuclear membrane protein XMAN2 |  |  | -1.3 | -1.3 |
| ID0AFF13AF06CM1 | CL460Contig1 | | A2A9F5 | | 3.00E-88 | | Nuclear migration protein |  |  | 1.3 | 1.4 |
| MpW-X-A07 | CL_MP_7295.3-34966614 | | Q7JZN0 | | 7.00E-06 | | Sec61β |  |  | 1.6 |  |
| ApHL3SD-III-F3 | CL87Contig1 | | Q5XUA5 | | 0 | | Sec61α |  |  |  | 1.7 |
| ID0AAK3YM09CM1 | CL7Contig4 | | Q7JZN0 | | 6.00E-28 | | Sec61β |  |  | 1.9 |  |
| ID0AFF8AH08CM1 | gi|82567142|gb|DV742832.1|DV742832 | | Q5TIE1 | | 2E-15 | | Solute carrier family |  |  | -1.3 | -1.4 |
| ID0AFF8CH07CM1 | CL126Contig1 | | Q9VJ43 | | 0 | | Sterol carrier protein x |  |  |  | -1.3 |
| ID0AAK4YI08CM1 | CL5657Contig1 | | Q29CT0 | | 8.00E-09 | | Apolipophorin |  |  | 1.5 |  |
| ApHL3SD-XIX-C8 | CL1078Contig2 | | Q9VPJ9 | | 1.00E-137 | | ATPase |  |  | -1.8 | -1.9 |
| ID0AAH2DC01ZM1 | CL391Contig1 | | Q9XZ63 | | 7.00E-45 | | ARP-like |  |  | 1.5 | 1.6 |
| ApHL3LD-IX-A2 | CL1221Contig1 | | Q17P45 | | 2E-13 | | Calcium binding protein |  |  | -1.4 |  |
| ID0AAK2YI19CM1 | CL39Contig1 | | Q8SY75 | | 2.00E-73 | | Calcium ion binding |  |  |  | -1.5 |
| ID0AAK5YM19CM1 | CL1Contig1351 | | Q7JYZ0 | | 3.00E-35 | | Calcium ion binding |  |  |  | 1.4 |
| ApDT-XXXII-B5 | CL6181Contig1 | | Q9VLC3 | | 2.00E-35 | | Ferroxidase |  |  | -1.3 | -1.3 |
| ID0AAK1YP04CM1 | CL779Contig1 | | Q9VY93 | | 2.00E-69 | | Metal ion binding |  |  | 1.6 | 2.2 |
| ID0AAK2YO02CM1 | CL1929Contig1 | | Q9VAF4 | | 2.00E-25 | | Mg ion binding |  |  | -1.4 |  |
| ApHL3LD-III-D3 | CL1959Contig1 | | O16158 | | 1.00E-55 | | Sarcoplasmic calcium-binding protein |  |  |  | 1.4 |
| ApAL3SD-VIII-E7 | CL6608Contig1 | | Q9VPE3 | | 4E-17 | | Zinc ion transmembrane transporter |  |  |  | 1.3 |
| **Defence and stress response** | | | | | | | | | | | |
| **ESTs** | **Contig** | | **Uniprot Accession** | | **E-value** | | **Gene Name** | **L4-G0** | **A-G0** | **L2-G1** | **L4-G1** |
|  |  | |  | |  | |  |  |  |  |  |
| ID0AAK9YI05CM1 | CL1767Contig1 | | Q7JUZ6 | | 2.00E-31 | | Heat shock protein binding |  |  | -1.5 |  |
| ID0AAG4AE12CM1 | CL238Contig1 | | A4V4C4 | | 0 | | Heat shock protein |  |  | 1.7 | 1.5 |
| MpW-XX-G05 | CL_MP_4111.3-34966941 | | P11147 | | 1.00E-14 | | Hsp 70 |  |  | 1.3 | 1.6 |
| ApAL3SD-IV-B11 | CL2253Contig1 | | Q9VX95 | | 1E-14 | | Hsp70 |  |  |  | -1.4 |
| ApHL3SD-XXX-D6 | CL1Contig1125 | | A8D4R5 | | 0 | | Hsp90 |  |  |  | 1.5 |
| ApHL3SD-XXXII-C4 | CL1Contig1212 | | Q57SQ8 | | 2E-16 | | Holin-like protein |  |  |  | 2.3 |
| ID0AAK5YB08CM1 | CL205Contig1 | | Q65Y02 | | 8.00E-72 | | Superoxide dismutase | 1.2 |  | -2.3 | 3.0 |
|  |  | |  | |  | |  |  |  |  |  |
| **Translation, transcription and replication** | | | | | | | | | | | |
| **ESTs** | **Contig** | | **Uniprot Accession** | | **E-value** | | **Gene Name** | **L4-G0** | **A-G0** | **L2-G1** | **L4-G1** |
|  |  | |  | |  | |  |  |  |  |  |
| ID0AAK2YI24CM1 | CL1316Contig1 | | Q7PCY7 | | 7.00E-19 | | DNA binding |  |  |  | 3.2 |
| ID0AAG15BF01CM1 | CL1589Contig1 | | Q16GN9 | | 1.00E-111 | | DNA primase |  |  |  | 1.8 |
| ID0AAK5YD14CM1 | CL265Contig2 | | Q8T8V5 | | 2.00E-33 | | Autoantigen-like |  |  | -1.3 |  |
| Ap_SDU2_3B04_SP6 | No Contig | | Q9W1V3 | | 8.00E-14 | | O-methyltransferase fibrillarin |  |  |  | 2.4 |
| ID0AAK10YG12CM1 | gi|86461307|gb|DY227179.1|DY227179 | | Q5TRZ9 | | 5E-11 | | Poly(rC) binding |  |  | -2.2 | -3.0 |
| ApHL3LD-VI-A12 | CL3854Contig1 | | Q9VTL6 | | 2.00E-33 | | RNA polymerase |  |  | 1.4 |  |
| ID0AAK1YH17CM1 | CL1Contig294 | | Q5UEN0 | | 4.00E-66 | | RNA polymerase |  |  |  | 1.3 |
| ID0AAK10YG06CM1 | CL6638Contig1 | | Q17CW3 | | 6E-12 | | mRNA binding |  |  | -2.6 |  |
| ID0AAG1BB12CM1 | CL1Contig30 | | Q4GXL8 | | 2.00E-97 | | Ribosomal protein |  |  |  | 1.3 |
| ID0AAG8AG08CM1 |  | | Q4GXL8 | | 2.00E-97 | | Ribosomal protein |  |  | 1.3 | 1.4 |
| ID0AFF6BH04CM1 |  | | Q4GXL8 | | 2.00E-97 | | Ribosomal protein |  |  |  | 1.5 |
| ApDT-V-c4 | CL47Contig1 | | Q0E8B5 | | 1.00E-113 | | Ribosomal protein |  |  | 1.4 | 1.5 |
| ApHL3LD-XIII-C4 |  | | Q0E8B5 | | 1.00E-113 | | Ribosomal protein |  |  | 1.3 |  |
| ApHL3LD-XIII-E6 |  | | Q0E8B5 | | 1.00E-113 | | Ribosomal protein |  |  | 1.3 | 1.4 |
| ApHL3SD-I-E4 |  | | Q0E8B5 | | 1.00E-113 | | Ribosomal protein |  |  | 1.3 |  |
| ID0AAH11BG08ZM1 |  | | Q0E8B5 | | 1.00E-113 | | Ribosomal protein |  |  | 1.3 |  |
| ID0AAK1YP12CM1 |  | | Q0E8B5 | | 1.00E-113 | | Ribosomal protein |  |  | 1.4 |  |
| ID0AAG7BC09CM1 |  | | Q0E8B5 | | 1.00E-113 | | Ribosomal protein |  |  | 1.7 |  |
| ID0AAK1YI19CM1 | CL73Contig1 | | A2I419 | | 0 | | Ribosomal protein |  |  |  | 1.7 |
| ID0AFF3BD06CM1 |  | | A2I419 | | 0 | | Ribosomal protein |  |  | 1.4 | 1.5 |
| ApHL3LD-XIV-A1 | CL1Contig1217 | | Q201X1 | | 6.00E-65 | | Ribosomal protein |  |  |  | 1.3 |
| ID0AAK5YJ01CM1 |  | | Q201X1 | | 6.00E-65 | | Ribosomal protein |  |  |  | 1.6 |
| Ap_SDD3_4A09_SP6 | CTG_AP_502.5-ID0AFF9AD06CM1 | | P48601 | | 1.00E-43 | | Proteasome 26S ATPase |  |  | 1.5 |  |
| ID0AFF11DC04CM1 | CL277Contig2 | | Q9NB22 | | 6.00E-50 | | EIF4E |  |  | 1.4 | 1.4 |
| ID0AAK1YG03CM1 | CL1Contig260 | | A4V101 | | 0 | | Elongation factor 2 |  |  | 1.3 | 1.3 |
| ID0AAK2YH07CM1 | CL392Contig2 | | Q9BPS1 | | 4.00E-59 | | Elongation factor 2 |  |  |  | 2.0 |
| ID0AAK1YP05CM1 | CL961Contig1 | | Q9V3P3 | | 3.00E-59 | | Proteasome regulator |  |  | 2.1 | 3.0 |
| ApHL3LD-XVII-F3 | CL201Contig1 | | Q16UH5 | | 2.00E-94 | | Proteasome subunit alpha |  |  | -2.0 | 2.0 |
| ID0AAK1YL08CM1 | CL1918Contig1 | | P55795 | | 8.00E-21 | | Ribonucleoprotein |  |  | 1.3 | 1.3 |
| ApAL3SD-XIV-C2 | CL71Contig1 | | Q56FG7 | | 1.00E-156 | | Ribosomal protein |  |  |  | 1.4 |
| ApDT-XXI-E4 | CL1Contig28 | | Q0PXW7 | | 1.00E-109 | | Ribosomal protein |  |  | 1.5 | 1.5 |
| ApHL3LD-III-G7 | CL1Contig1012 | | A2IAA5 | | 4.00E-67 | | Ribosomal protein |  |  | -2.4 |  |
| ApHL3LD-V-B1 | CL10Contig1 | | Q201V7 | | 2.00E-76 | | Ribosomal protein |  |  | -2.8 | -2.5 |
| ApHL3LD-XIII-G8 | CL1Contig743 | | Q201W1 | | 4.00E-60 | | Ribosomal protein |  |  | 1.3 | 1.8 |
| ApHL3SD-XX-D12 | CL1Contig1100 | | A8CW94 | | 4.00E-22 | | Ribosomal protein |  |  |  | 1.4 |
| ApHL3SD-XXIX-B5 | CL1Contig712 | | Q4LAX8 | | 1.00E-35 | | Ribosomal protein |  |  | 1.3 | 1.4 |
| ID0AAG10DH01CM1 | CL1Contig417 | | Q4VM05 | | 1.00E-104 | | Ribosomal protein |  |  | 1.3 | 1.5 |
| ID0AAG11AA07CM1 | CL1Contig1124 | | Q201X6 | | 4.00E-74 | | Ribosomal protein |  |  | 1.3 | 1.3 |
| ID0AAG11BD06CM1 | CL351Contig1 | | Q201Z2 | | 3.00E-64 | | Ribosomal protein |  |  | 1.3 |  |
| ID0AAG11CB09CM1 | CL8Contig2 | | Q201X3 | | 2.00E-34 | | Ribosomal protein |  |  | 1.3 | 1.4 |
| ID0AAG11DB12CM1 | CL567Contig1 | | Q201U8 | | 4.00E-21 | | Ribosomal protein |  |  | 1.5 | 1.4 |
| ID0AAG11DG11CM1 | CL1Contig104 | | Q8T3U2 | | 2.00E-73 | | Ribosomal protein |  |  | 1.3 | 1.3 |
| ID0AAG12AD02CM1 | CL1Contig1094 | | Q4GXS1 | | 4.00E-71 | | Ribosomal protein |  |  | 1.3 | 1.3 |
| ID0AAG12BH09CM1 | CL1Contig850 | | Q56FE7 | | 6.00E-70 | | Ribosomal protein |  |  | 1.4 | 1.5 |
| ID0AAG12DE12CM1 | CL1Contig161 | | Q4GXA3 | | 5.00E-46 | | Ribosomal protein |  |  |  | 1.3 |
| ID0AAG12DF11CM1 | CL8Contig4 | | A2I448 | | 1.00E-101 | | Ribosomal protein |  |  |  | 1.4 |
| ID0AAG13DD06CM1 | CL1Contig840 | | Q4GXG0 | | 4.00E-62 | | Ribosomal protein |  |  | 1.3 | 1.3 |
| ID0AAG15DF01CM1 | CL5Contig3 | | Q6EV05 | | 1.00E-105 | | Ribosomal protein |  |  | 1.3 | 1.3 |
| ID0AAG1BF02CM1 | CL1Contig273 | | Q201V9 | | 4.00E-55 | | Ribosomal protein |  |  | 1.3 | 1.4 |
| ID0AAG1BH04CM1 | CL1Contig1132 | | A2I405 | | 2.00E-78 | | Ribosomal protein |  |  | 1.5 | 1.5 |
| ID0AAG2DA01CM1 | CL47Contig2 | | A2I3W7 | | 1.00E-118 | | Ribosomal protein |  |  | 1.3 |  |
| ID0AAG3CG02CM1 | CL1Contig374 | | Q4GXJ6 | | 2.00E-87 | | Ribosomal protein |  |  | 1.4 | 1.5 |
| ID0AAG4DF02CM1 | CL140Contig1 | | A8E7A3 | | 6.00E-42 | | Ribosomal protein |  |  | 1.3 | 1.3 |
| ID0AAG7AH11CM1 | CL1Contig1177 | | Q7PL67 | | 1.00E-117 | | Ribosomal protein |  |  |  | 1.3 |
| ID0AAG8BF06CM1 | CL26Contig1 | | Q9V9W3 | | 7.00E-37 | | Ribosomal protein |  |  | 1.4 | 1.4 |
| ID0AAK10YA14CM1 | CL1Contig862 | | Q201W0 | | 1.00E-66 | | Ribosomal protein |  |  |  | 1.8 |
| ID0AAK10YK08CM1 | gi|86461392|gb|DY227264.1|DY227264 | | Q0PXW7 | | 8E-11 | | Ribosomal protein |  |  |  | 2.4 |
| ID0AAK10YK09CM1 | CL1Contig250 | | Q4GXF2 | | 9.00E-26 | | Ribosomal protein |  |  | 4.1 | 9.7 |
| ID0AAK1YC12CM1 | CL198Contig2 | | Q4GXP0 | | 5.00E-30 | | Ribosomal protein |  |  | 1.9 | 2.2 |
| ID0AAK1YI12CM1 | gi|86461704|gb|DY227576.1|DY227576 | | Q4TTD7 | | 2E-11 | | Ribosomal protein |  |  | 1.5 | 2.2 |
| ID0AAK1YL12CM1 | CL73Contig2 | | A2I419 | | 0 | | Ribosomal protein |  |  | 1.4 | 1.7 |
| ID0AAK1YL23CM1 | CL1015Contig1 | | Q16ML4 | | 5.00E-85 | | Ribosomal protein |  |  |  | 2.4 |
| ID0AAK2YF18CM1 | gi|86461978|gb|DY227850.1|DY227850 | | Q201V7 | | 1.00E-58 | | Ribosomal protein |  |  | 1.8 | 2.0 |
| ID0AAK2YK13CM1 | CL1Contig1127 | | Q201V9 | | 3.00E-23 | | Ribosomal protein |  |  | -3.7 |  |
| ID0AAK3YF22CM1 | CL1Contig1000 | | A2I497 | | 1.00E-86 | | Ribosomal protein |  |  | 1.8 | 2.0 |
| ID0AAK4YC07CM1 | CL1Contig835 | | Q6EUY9 | | 2.00E-53 | | Ribosomal protein |  |  | 1.6 | 1.9 |
| ID0AAK4YL22CM1 | CL174Contig1 | | Q201Z1 | | 1.00E-44 | | Ribosomal protein |  |  |  | 1.4 |
| ID0AAK5YG01CM1 | CL1Contig805 | | Q4GXG8 | | 2.00E-57 | | Ribosomal protein |  |  | 1.5 | 2.6 |
| ID0AAK5YG05CM1 | CL86Contig2 | | A6YPH7 | | 1.00E-130 | | Ribosomal protein |  |  | 1.6 |  |
| ID0AAK6YL19CM1 | CL180Contig2 | | Q201X4 | | 2.00E-71 | | Ribosomal protein |  |  | 1.3 | 1.3 |
| ID0AAK6YN13CM1 | CL198Contig1 | | Q4GXP0 | | 6.00E-30 | | Ribosomal protein |  |  | 1.3 | 1.3 |
| ID0AAK8YN10CM1 | CL1Contig36 | | A2I410 | | 6.00E-40 | | Ribosomal protein |  |  |  | 1.3 |
| ID0AAK9YL17CM1 | CL1Contig496 | | Q201W9 | | 1.00E-152 | | Ribosomal protein |  |  | 1.3 | 1.3 |
| ID0AFF10DB03CM1 | CL363Contig1 | | A1Z9J6 | | 3.00E-24 | | Ribosomal protein |  |  | 1.2 | 1.3 |
| ID0AFF12DE07CM1 | CL1Contig212 | | Q1ZZQ9 | | 5.00E-93 | | Ribosomal protein |  |  | 1.3 |  |
| ID0AFF13CC01CM1 | CL1Contig363 | | Q4GXE1 | | 3.00E-45 | | Ribosomal protein |  |  |  | 1.3 |
| ID0AFF13DE10CM1 | CL1Contig383 | | Q201V9 | | 3.00E-55 | | Ribosomal protein |  |  | 1.3 |  |
| ID0AFF14AB05CM1 | CL1Contig408 | | Q4GXE1 | | 2.00E-45 | | Ribosomal protein |  |  | 1.3 | 1.3 |
| ID0AFF1AG07CM1 | CL1Contig982 | | Q56FF3 | | 8.00E-63 | | Ribosomal protein |  |  | 1.3 |  |
| ID0AFF4DC11CM1 | gi|82565941|gb|DV741631.1|DV741631 | | Q201W1 | | 5.00E-21 | | Ribosomal protein |  |  | 1.3 |  |
| ID0AFF8BG01CM1 | CL1Contig713 | | A2I3U5 | | 4.00E-88 | | Ribosomal protein |  |  | 1.3 |  |
| MpW-I-B01 | CTG_MP_513.3-34965994 | | Q1ZZQ1 | | 2.00E-17 | | Ribosomal protein |  |  | 1.3 | 1.4 |
| MpW-I-C02 | CL_MP_6463.3-34965989 | | Q6F442 | | 1.00E-12 | | Ribosomal protein |  |  | 1.3 | 1.4 |
| MpW-I-C06 | CL_MP_5226.3-34965991 | | Q4GXC3 | | 2.00E-05 | | Ribosomal protein |  |  | 1.4 | 1.5 |
| MpW-I-F12 | CTG_MP_2787.3-34966135 | | Q56FL4 | | 2.00E-15 | | Ribosomal protein |  |  | 1.3 |  |
| MpW-I-G08 | CTG_MP_14.3-34597422 | | Q4GXE1 | | 1.00E-44 | | Ribosomal protein |  |  | 1.3 |  |
| MpW-I-G10 | CTG_MP_2599.3-34966099 | | A2I448 | | 5.00E-23 | | Ribosomal protein |  |  | 1.3 | 1.4 |
| MpW-III-E04 | CTG_MP_79.3-34598199 | | Q201Z1 | | 9.00E-42 | | Ribosomal protein |  |  | 1.3 |  |
| MpW-IV-G12 | CL_MP_4071.3-34965999 | | Q801G5 | | 2.00E-16 | | Ribosomal protein |  |  | 1.3 |  |
| MpW-IV-H03 | CTG_MP_31.3-34598103 | | Q6EUY9 | | 2.00E-52 | | Ribosomal protein |  |  | 1.3 |  |
| MpW-IX-A11 | CTG_MP_58.3-34598036 | | Q5XUC7 | | 2.00E-155 | | Ribosomal protein |  |  | 1.3 | 1.3 |
| MpW-IX-B01 | CTG_MP_23.3-34597783 | | A2I3Z8 | | 3.00E-87 | | Ribosomal protein |  |  | 1.3 | 1.3 |
| MpW-IX-B03 | CTG_MP_97.3-34966432 | | Q0PXV9 | | 8.00E-17 | | Ribosomal protein |  |  | 1.3 | 1.4 |
| MpW-V-B06 | CL_MP_6430.3-34966132 | | Q201Y6 | | 2.00E-19 | | Ribosomal protein |  |  |  | 1.3 |
| MpW-V-C05 | CTG_MP_17.3-34597554 | | Q66SW3 | | 6.00E-66 | | Ribosomal protein |  |  | 1.3 | 1.3 |
| MpW-V-D12 | CL_MP_8776.3-34966136 | | Q4GXK4 | | 9.00E-24 | | Ribosomal protein |  |  | 1.3 |  |
| MpW-V-G02 | CL_MP_9387.3-34966262 | | Q6XI13 | | 7.00E-13 | | Ribosomal protein |  |  | 1.4 | 1.6 |
| MpW-VI-A03 | CL_MP_7319.3-34966230 | | P62244 | | 5.00E-17 | | Ribosomal protein |  |  | 1.4 |  |
| MpW-VI-C01 | CTG_MP_26.3-34597332 | | Q0PXY3 | | 9.00E-77 | | Ribosomal protein |  |  | 1.4 | 1.4 |
| MpW-VI-D10 | CTG_MP_72.3-34597413 | | Q66SW3 | | 3.00E-33 | | Ribosomal protein |  |  | 1.3 |  |
| MpW-VI-E11 | CTG_MP_134.3-34597522 | | P41042 | | 4.00E-122 | | Ribosomal protein |  |  | 1.3 |  |
| MpW-VII-D12 | CL_MP_4024.3-34966274 | | Q174U2 | | 1.00E-15 | | Ribosomal protein |  |  | 1.3 |  |
| MpW-VIII-A04 | CL_MP_4368.3-34966374 | | Q0PXY2 | | 1.00E-11 | | Ribosomal protein |  |  | 1.4 | 1.4 |
| MpW-VIII-B01 | CTG_MP_26.3-34597332 | | Q0PXY3 | | 9.00E-77 | | Ribosomal protein |  |  | 1.3 | 1.3 |
| MpW-X-A08 | CL_MP_5440.3-34966633 | | A6YPK3 | | 1.00E-23 | | Ribosomal protein |  |  | 1.3 | 1.4 |
| MpW-XII-A01 | CTG_MP_16.3-34597356 | | A2I496 | | 2.00E-88 | | Ribosomal protein |  |  | 1.4 |  |
| MpW-XII-B01 | CTG_MP_99.3-34597308 | | A6YPH7 | | 2.00E-129 | | Ribosomal protein |  |  | 1.3 | 1.4 |
| MpW-XII-G04 | CTG_MP_28.3-34597322 | | A6YPQ3 | | 2.00E-40 | | Ribosomal protein |  |  |  | 1.3 |
| MpW-XIII-B05 | CTG_MP_51.3-34598741 | | Q9W237 | | 1.00E-68 | | Ribosomal protein |  |  | 1.3 | 1.4 |
| MpW-XIII-D01 | CTG_MP_1773.3-34966413 | | Q201W7 | | 8.00E-68 | | Ribosomal protein |  |  | 1.4 | 1.5 |
| MpW-XIII-E03 | CTG_MP_1223.3-34966331 | | Q0PXX8 | | 1.00E-72 | | Ribosomal protein |  |  | 1.3 | 1.3 |
| MpW-XIII-G05 | CTG_MP_3840.3-34966399 | | Q5F346 | | 7.00E-09 | | Ribosomal protein |  |  | 1.3 | 1.5 |
| MpW-XIX-G12 | CL_MP_9028.3-34966895 | | Q1HR65 | | 6.00E-13 | | Ribosomal protein |  |  |  | 1.4 |
| MpW-XV-C03 | CL_MP_6790.3-34966677 | | Q4GXI2 | | 6.00E-31 | | Ribosomal protein |  |  | 1.3 |  |
| MpW-XV-E04 | CL_MP_8521.3-34966746 | | Q4GXP7 | | 2.00E-07 | | Ribosomal protein |  |  | 1.3 |  |
| MpW-XVI-A07 | CL_MP_4407.3-34966771 | | A2IAE6 | | 2.00E-09 | | Ribosomal protein |  |  | 1.3 | 1.5 |
| MpW-XVI-G06 | CL_MP_9360.3-34966725 | | Q4PM23 | | 9.00E-14 | | Ribosomal protein |  |  | 1.3 | 1.3 |
| MpW-XVI-H03 | CTG_MP_1223.3-34966331 | | Q0PXX8 | | 1.00E-72 | | Ribosomal protein |  |  | 1.3 |  |
| MpW-XVII-A03 | CTG_MP_92.3-34597650 | | A2I469 | | 3.00E-31 | | Ribosomal protein |  |  | 1.3 | 1.4 |
| MpW-XVII-G06 | CL_MP_6868.3-34966896 | | A6N9M1 | | 2.00E-20 | | Ribosomal protein |  |  |  | 1.3 |
| MpW-XVIII-D11 | CTG_MP_1877.3-34966054 | | A2IAE1 | | 1.00E-22 | | Ribosomal protein |  |  | 1.3 |  |
| MpW-XVIII-G08 | CL_MP_8612.3-34966907 | | Q4GXN1 | | 5.00E-27 | | Ribosomal protein |  |  | 1.3 | 1.3 |
| MpW-XX-F12 | CTG_MP_23.3-34597783 | | A2IAE1 | | 3.00E-87 | | Ribosomal protein |  |  | 1.3 | 1.4 |
| MpW-XX-G10 | CL_MP_6928.3-34966934 | | Q56FH8 | | 1.00E-18 | | Ribosomal protein |  |  | 1.3 |  |
| ID0AFF4DC03CM1 | CL74Contig3 | | Q1ZZQ5 | | 2.00E-57 | | Ribosomal protein |  |  | 1.3 | 1.4 |
| ID0AAK1YH16CM1 | CL2382Contig1 | | Q8IQI9 | | 1.00E-59 | | Ribosome |  |  | -1.3 | -1.4 |
| ID0AAK4YB21CM1 | CL556Contig2 | | Q9VU43 | | 1.00E-42 | | Ribosome |  |  |  | 1.3 |
| ApHL3LD-IX-A10 | CL1240Contig1 | | Q9VX98 | | 2.00E-63 | | Translation initiation factor |  |  | -1.4 |  |
| ApHL3SD-II-D12 | CL3866Contig1 | | Q8CFW0 | | 6.00E-20 | | Translation initiation factor |  |  |  | 1.3 |
| ID0AAG9DB12CM1 | CL92Contig2 | | Q9VN25 | | 0 | | Translation initiation factor |  |  |  | 1.5 |
| ID0AAK3YE09CM1 | CL1158Contig1 | | Q9W4X7 | | 8.00E-65 | | translation initiation factor |  |  |  | 1.3 |
| ID0AFF1DA10CM1 | CL206Contig1 | | Q7K550 | | 9.00E-20 | | Translation initiation factor |  |  | 1.3 |  |
| ID0AAK5YP13CM1 | CL1Contig1011 | | A2I3U4 | | 7E-15 | | Ribosomal protein |  |  | 1.5 | 2.3 |
|  |  | |  | |  | |  |  |  |  |  |
| **Cuticle structure and synthesis** | | | | | | | | | | | |
| **ESTs** | **Contig** | | **Uniprot Accession** | | **E-value** | | **Gene Name** | **L4-G0** | **A-G0** | **L2-G1** | **L4-G1** |
|  |  | |  | |  | |  |  |  |  |  |
| ApHL3SD-XXXII-E11 | CL1Contig163 | | B2DBF7 | | 1.00E-11 | | Cuticular protein |  |  | 1.3 | 1.7 |
| ID0AAK7YE01CM1 |  | | B2DBF7 | | 1.00E-11 | | Cuticular protein |  |  | -5.8 | 1.3 |
| ApHL3SD-XXXI-F9 | CL1Contig281 | | A8DRW0 | | 4.00E-24 | | Cuticular protein |  | 1.4 | -3.0 | -2.8 |
| ID0AAK1YE23CM1 |  | | A8DRW0 | | 4.00E-24 | | Cuticular protein |  | 1.8 | -5.0 | -8.3 |
| ID0AAK1YB23CM1 | CL1Contig856 | | Q45V95 | | 2.00E-51 | | Cuticular protein |  |  | -3.7 | -6.3 |
| ID0AAK5YD19CM1 |  | | Q45V95 | | 2.00E-51 | | Cuticular protein |  |  | -4.0 | -8.0 |
| ID0AAK2YH04CM1 | CL1Contig954 | | Q45V96 | | 4.00E-06 | | Cuticular protein |  |  |  | 13.3 |
| ID0AAK9YI10CM1 |  | | Q45V96 | | 4.00E-06 | | Cuticular protein |  |  |  | 8.9 |
| ApHL3SD-XXVIII-D1 | CL667Contig1 | | Q5TWJ2 | | 2.00E-27 | | Cuticular protein | -1.5 |  | -1.5 | -6.0 |
| ID0AFF12BC01CM1 |  | | Q5TWJ2 | | 2.00E-27 | | Cuticular protein |  |  | -1.7 | -6.8 |
| ApAL3SD-VI-A11 | CL17Contig2 | | Q95V16 | | 3.00E-23 | | Cuticular protein |  |  | -1.9 | -8.3 |
| ID0AAK4YB22CM1 |  | | Q95V16 | | 3.00E-23 | | Cuticular protein | -2.3 |  | -3.6 | -112.5 |
| ApHL3LD-XIII-A9 | CL1Contig1228 | | Q45V96 | | 8.00E-10 | | Cuticular protein |  |  | -1.6 | 1.4 |
| ID0AAK3YH08CM1 |  | | Q45V96 | | 8.00E-10 | | Cuticular protein |  |  | -2.1 | 2.2 |
| ApHL3SD-XXVII-B5 | CL1Contig1247 | | Q95V16 | | 5.00E-25 | | Cuticular protein | -1.8 |  | -4.7 | -14.7 |
| ID0AAK2YK14CM1 |  | | Q95V16 | | 5.00E-25 | | Cuticular protein | -3.1 |  | -6.5 | -125.8 |
| ID0AAH12AA08ZM1 | CL1150Contig1 | | Q9VR79 | | 3.00E-56 | | Chitin binding |  |  |  | -1.4 |
| ApAL3SD-II-D1 | CL67Contig1 | | Q45V95 | | 1.00E-38 | | Cuticular protein |  |  | -2.4 | -6.1 |
| ApAL3SD-III-F4 | CL1Contig476 | | Q5TR22 | | 1.00E-30 | | Cuticular protein |  |  | 1.7 | -1.9 |
| ApAL3SD-XII-A2 | CL568Contig1 | | Q86GL3 | | 1.00E-25 | | Cuticular protein |  |  | 1.5 |  |
| ApAL3SD-XIX-D8 | CL1Contig631 | | Q95V16 | | 1.00E-20 | | Cuticular protein | -1.6 |  | -2.9 | -4.6 |
| ApAL3SD-XVI-G10 | CL1Contig454 | | Q95V16 | | 1.00E-43 | | Cuticular protein | -1.8 |  | -4.9 | -19.6 |
| ApAL3SD-XVIII-B9 | CL778Contig1 | | Q95V16 | | 2.00E-25 | | Cuticular protein |  |  |  | -2.6 |
| ApAL3SD-XX-F7 | CL1Contig1146 | | Q95V16 | | 8.00E-26 | | Cuticular protein |  |  | -2.2 | -4.6 |
| ApHL3SD-I-D1 | CL1Contig490 | | Q95V16 | | 1.00E-22 | | Cuticular protein |  |  | -2.4 | -3.0 |
| ID0AAK1YD01CM1 | CL12Contig1 | | Q45V94 | | 2.00E-53 | | Cuticular protein |  | 2.3 | -4.1 | 2.2 |
| ID0AAK1YF06CM1 | CL1Contig690 | | Q95V16 | | 2.00E-27 | | Cuticular protein | -3.5 |  | -9.3 | -38.4 |
| ID0AAK2YA19CM1 | CL1Contig707 | | Q95V16 | | 3.00E-21 | | Cuticular protein |  |  |  | -55.0 |
| ID0AAK3YF14CM1 | CL1Contig1346 | | Q95V16 | | 4.00E-21 | | Cuticular protein | -2.0 |  | -4.4 | -49.2 |
| ID0AAK4YG05CM1 | CL17Contig3 | | Q95V16 | | 3.00E-23 | | Cuticular protein | -2.0 |  | -3.0 | -13.8 |
| ID0AAK4YH18CM1 | CL1Contig1043 | | Q95V16 | | 1.00E-24 | | Cuticular protein | -3.9 | 3.1 | -7.8 |  |
| ID0AAK4YI23CM1 | CL1Contig279 | | Q7QIK6 | | 4E-13 | | Cuticular protein |  |  |  | 1.8 |
| ID0AAK4YM05CM1 | CL1Contig990 | | Q95V16 | | 8.00E-27 | | Cuticular protein | -2.7 |  | -7.1 |  |
| ID0AAK4YM19CM1 | gi|86462801|gb|DY228673.1|DY228673 | | Q45V95 | | 4.00E-24 | | Cuticular protein |  |  | -4.5 | -9.2 |
| ID0AAK4YO19CM1 | CL1115Contig1 | | A8DRW0 | | 9E-12 | | Cuticular protein |  |  |  | -3.4 |
| ID0AAK5YG20CM1 | CL1Contig1205 | | Q45V96 | | 4.00E-10 | | Cuticular protein |  |  | -4.9 |  |
| ID0AAK5YN01CM1 | CL1Contig858 | | Q95V16 | | 8.00E-26 | | Cuticular protein | -3.5 |  | -9.9 | -500.3 |
| ID0AAK6YG06CM1 | CL1Contig1141 | | Q45V96 | | 4.00E-06 | | Cuticular protein |  |  |  | 4.8 |
| ID0AAK8YB10CM1 | CL1Contig957 | | B2DBF7 | | 2.00E-13 | | Cuticular protein |  |  | -2.9 | 3.3 |
| ID0AAK8YK19CM1 | gi|86464122|gb|DY229994.1|DY229994 | | Q9VB82 | | 3.00E-28 | | Cuticular protein |  |  | -6.7 | -5.5 |
| ID0AAK9YM18CM1 | CL1Contig261 | | Q7QIK6 | | 9.00E-20 | | Cuticular protein |  |  | -2.8 |  |
| MpW-IX-D05 | CL_MP_4859.3-34966509 | | Q86GK9 | | 1.00E-09 | | Cuticular protein |  |  | -3.3 | -27.5 |
| MpW-VI-B06 | CTG_MP_8.3-34597733 | | Q45V96 | | 1.00E-07 | | Cuticular protein |  |  |  | 3.2 |
| MpW-XIV-H07 | CL_MP_6768.3-34966583 | | Q45V95 | | 7.00E-15 | | Cuticular protein |  |  | -1.4 | -1.8 |
| ApAL3SD-II-E8 | CL1Contig272 | | Q4LEQ7 | | 8E-16 | | Glycin rich protein |  |  | -1.3 | -2.1 |
| ApAL3SD-XVII-E4 | CL1Contig473 | | Q4LEQ7 | | 1.00E-26 | | Glycin rich protein |  |  |  | -3.8 |
| ID0AAK6YC06CM1 | CL1Contig1088 | | Q4LEQ7 | | 2.00E-06 | | Glycine rich protein |  |  | -2.4 | 2.2 |
| ID0AAK5YI04CM1 | CL5271Contig1 | | Q9VEJ9 | | 9.00E-36 | | Peroxidase activity |  |  | 1.5 | -1.3 |
|  |  | |  | |  | |  |  |  |  |  |
| **Cytoskeleton and vesicles** | | | | | | | | | | | |
| **ESTs** | **Contig** | **Uniprot Accession** | | **E-value** | | **Gene Name** | | **L4-G0** | **A-G0** | **L2-G1** | **L4-G1** |
|  |  |  | |  | |  | |  |  |  |  |
| ApHL3SD-V-E10 | CL43Contig2 | Q6XHQ1 | | 4.00E-64 | | Actin binding | |  |  |  | 1.3 |
| ID0AAK3YO08CM1 | CL1Contig1135 | Q1HR53 | | 0 | | Alpha 2-tubulin | |  |  |  | 1.6 |
| ID0AAK1YH02CM1 | CL9681Contig1 | A1ZAJ2 | | 4.00E-80 | | Kinesin Dunc 10-4A | |  |  | -1.3 | -1.4 |
| ID0AAK10YP05CM1 | CL7245Contig1 | Q17EW2 | | 3.00E-24 | | Laminin | |  |  | -1.3 | -1.4 |
| ID0AAK5YH06CM1 | CL1Contig1391 | Q178Y4 | | 0 | | Myosin heavy chain | |  |  | -1.3 | -1.3 |
| ApAL3SD-XVIII-A6 | CL289Contig1 | Q297S0 | | 5.00E-20 | | Tubulin folding | |  |  |  | 1.3 |
| ApHL3SD-XII-C9 | CL2613Contig1 | Q17208 | | 7E-11 | | Collagen | |  |  | -1.4 |  |
| ID0AAK10YE11CM1 |  | Q17208 | | 7E-11 | | Collagen | |  |  | 1.6 |  |
|  |  |  | |  | |  | |  |  |  |  |
| **Development and signalization** | | | | | | | | | | | |
| **ESTs** | **Contig** | **Uniprot Accession** | | **E-value** | | **Gene Name** | | **L4-G0** | **A-G0** | **L2-G1** | **L4-G1** |
|  |  |  | |  | |  | |  |  |  |  |
| ID0AAK3YJ21CM1 | CL922Contig1 | Q9VPX7 | | 2.00E-80 | | Adenylate cyclase binding - Capulet | |  |  |  | 1.7 |
| ApHL3LD-XVIII-H4 | CL903Contig1 | Q7KY08 | | 0 | | Argonaute-1 | |  | 1.2 | -1.4 |  |
| ID0AAK3YL11CM1 | CL329Contig1 | Q9VXQ5 | | 0 | | Tcp-1ζ | |  |  | 1.3 | 1.4 |
| ID0AAK9YH15CM1 | gi|86464392|gb|DY230264.1|DY230264 | Q8MQJ9 | | 4.00E-07 | | Brain tumor protein | |  |  | -1.7 |  |
| Ap_SDD3_6A12_SP6 | No Contig | A4V0I0 | | 7.00E-08 | | cAMP-dependent protein kinase 1 | |  |  | 1.4 |  |
| ApHL3LD-XVIII-H12 | CL4194Contig1 | Q9VDF4 | | 2.00E-91 | | Cortactin | |  |  |  | 1.8 |
| ID0AAH9DE04ZM1 | CL1Contig140 | A4V4L8 | | 2.00E-81 | | Dorsal switch protein Dsp2 | |  |  | 1.4 |  |
| ApAL3SD-XVI-A11 | CL1031Contig1 | Q7Q9Y9 | | 3.00E-49 | | Bx42 | |  |  |  | -1.7 |
| ApAL3SD-XII-H10 | gi|47514504|gb|CN749507.1|CN749507 | Q172M3 | | 5E-11 | | Not1 | |  |  |  | 2.2 |
| ID0AAK2YE07CM1 | CL5026Contig1 | A4V0B5 | | 6.00E-84 | | Nervana 2 | |  |  |  | -1.3 |
| ID0AAK4YP09CM1 | CL1Contig92 | O97121 | | 1.00E-136 | | Ecdysteroid-inducible polypeptide EIP40 | |  |  | -1.8 |  |
| ID0AAK3YD07CM1 | CL4099Contig1 | A8E775 | | 8.00E-79 | | Arrestin-2 | |  |  |  | 1.4 |
| ApHL3LD-XI-G11 | gi|47518059|gb|CN753062.1|CN753062 | Q9VN82 | | 4.00E-44 | | Canoe | |  |  | 1.4 | 1.5 |
| ID0AAK1YG23CM1 | CL6069Contig1 | Q28DE3 | | 7E-12 | | DEP domain containing | |  |  | -1.4 | -1.6 |
| ApHL3LD-III-F10 | CL1091Contig1 | Q7PNV7 | | 1.00E-27 | | Dreg5 | |  |  |  | 2.6 |
| ApAL3SD-XIV-E3 | CL3473Contig1 | Q9VN68 | | 5.00E-48 | | Farp1 protein | |  |  | -1.3 | -1.4 |
| MpW-VI-C02 | CL_MP_8273.3-34966339 | Q5XUC3 | | 4.00E-14 | | Protein kinase c receptor | |  |  | 1.3 | 1.5 |
| ApAL3SD-XX-C9 | CL505Contig1 | Q9V3Y3 | | 6E-14 | | Hdd11 | |  |  | -1.8 | -2.1 |
| ID0AAK3YL19CM1 | CL7746Contig1 | Q9V3C7 | | 5E-15 | | Inhibitor-2 | |  |  | 5.8 | 29.9 |
| ID0AAK9YI18CM1 | gi|86464415|gb|DY230287.1|DY230287 | Q17IL5 | | 3.00E-43 | | Insulin-like receptor | |  |  | -1.5 | -1.6 |
| ApHL3SD-III-H8 | CL323Contig2 | Q8CGB9 | | 4.00E-78 | | Insulin-degrading enzyme | |  |  | 1.3 | 1.3 |
| ID0AFF13DE09CM1 | CL5160Contig1 | Q9VBY8 | | 1.00E-54 | | Dunc-13-4A | |  |  | 1.3 | 1.3 |
| ID0AAK1YI09CM1 | CL2104Contig1 | A1ZAX8 | | 7.00E-42 | | Odorant binding | |  |  | -1.3 |  |
| ID0AAK6YI01CM1 | gi|86463377|gb|DY229249.1|DY229249 | A4UZI6 | | 4.00E-78 | | Ras-like GTP-binding protein Rho1 | |  |  |  | 5.1 |
| ID0AAK4YE17CM1 | gi|86462629|gb|DY228501.1|DY228501 | A1Z7S0 | | 4.00E-34 | | Receptor mediated endocytosis 8 | |  |  |  | 1.3 |
| ID0AAG11AD08CM1 | CL1Contig632 | Q5XUC3 | | 0 | | Receptor of activated protein kinase | |  |  | 1.3 | 1.4 |
| ID0AAK5YN04CM1 | CL1701Contig1 | Q0N2R9 | | 4.00E-06 | | Signal recognition particle | |  |  | 1.3 | 1.4 |
| MpW-I-A02 | CL_MP_5075.3-34965986 | Q29E72 | | 7.00E-07 | | Signal recognition particle 19 kDa | |  |  |  | -1.4 |
| ID0AAK1YM11CM1 | CL117Contig1 | Q9NAZ4 | | 4.00E-20 | | Neural Lazarillo | |  |  | 17.1 | 15.4 |
| ApHL3LD-IV-C3 | CL1Contig1089 | Q9NGZ2 | | 6.00E-49 | | Wunen | |  |  |  | 4.3 |
| ApHL3LD-XVII-G10 | CL94Contig1 | Q8T410 | | 1.00E-121 | | Calnexin | |  |  | 1.5 | 6.5 |
| ID0AAK5YD04CM1 | CL33Contig1 | Q9U916 | | 1.00E-151 | | Calreticulin | |  |  | 2.4 | 2.5 |
| ApHL3LD-XVII-H7 | CL425Contig1 | Q3YMW0 | | 2E-16 | | Ecdysone inducible protein L2 | |  |  | -1.5 | -1.5 |
| ApAL3SD-V-A1 | CL2227Contig1 | Q6NS86 | | 4E-15 | | Zinc finger protein | |  |  |  | 1.7 |
| ApHL3LD-IX-D8 | CL5507Contig1 | Q6R5P6 | | 5.00E-31 | | Zinc finger protein | |  |  |  | 3.1 |
| ApHL3LD-XII-C4 | gi|47518073|gb|CN753076.1|CN753076 | Q5QKN3 | | 3.00E-48 | | Zinc finger protein | |  |  | -2.5 | -4.7 |
| ID0AAG13BB02CM1 | CL1Contig978 | Q3L234 | | 2.00E-28 | | Zinc finger protein | |  |  | 1.6 | 1.5 |
|  |  |  | |  | |  | |  |  |  |  |
| **Orphan genes or transcripts homologous to hypothetical proteins** | | | | | | | | | | | |
|  |  |  | |  | |  | |  |  |  |  |
| **ESTs** | **Contig** | **Uniprot Accession** | | **E-value** | | **Gene Name** | | **L4-G0** | **A-G0** | **L2-G1** | **L4-G1** |
|  |  |  | |  | |  | |  |  |  |  |
| ID0AAK10YJ12CM1 | CL1Contig1387 | / | | / | | No hit | |  |  | -1.3 |  |
| ID0AAK10YO22CM1 |  | / | | / | | No hit | |  |  |  | 5.5 |
| ApHL3SD-XIV-B3 | CL1Contig47 | / | | / | | No hit | |  |  |  | 1.4 |
| ID0AAK2YE09CM1 |  | / | | / | | No hit | |  |  |  | 13.2 |
| ApAL3SD-XII-F9 | CL1Contig585 | / | | / | | No hit | |  |  |  | 2.9 |
| ID0AAK10YE03CM1 |  | / | | / | | No hit | |  |  |  | 6.1 |
| ApAL3SD-IV-H8 | CL3823Contig1 | / | | / | | No hit | |  |  | 1.5 | 2.3 |
| ApAL3SD-VII-F1 |  | / | | / | | No hit | |  |  | 1.7 | 2.4 |
| ApAL3SD-II-H8 | CL1337Contig1 | / | | / | | No hit | |  |  | 1.6 | 2.5 |
| ID0AAK6YA17CM1 |  | / | | / | | No hit | |  |  | 2.3 | 3.4 |
| ApAL3SD-II-D4 | CL1Contig1239 | / | | / | | No hit | |  |  |  | 1.8 |
| ApAL3SD-XII-C7 |  | / | | / | | No hit | |  |  | -1.3 | 1.5 |
| ID0AAK1YM06CM1 |  | / | | / | | No hit | |  |  |  | 4.5 |
| ApAL3SD-XX-D10 | CL1Contig1240 | / | | / | | No hit | |  |  |  | 2.5 |
| ApHL3SD-XI-F10 |  | / | | / | | No hit | |  |  |  | 1.7 |
| ID0AAK10YP03CM1 | CL1Contig1377 | / | | / | | No hit | |  |  |  | 1.3 |
| ID0AAK1YM02CM1 |  | / | | / | | No hit | |  |  |  | 2.3 |
| ApAL3SD-I-A12 | CL51Contig1 | / | | / | | No hit | |  |  | 1.6 |  |
| ApAL3SD-II-D5 | gi|47514020|gb|CN749023.1|CN749023 | / | | / | | No hit | |  |  |  | 1.7 |
| ApAL3SD-II-D9 | gi|47514023|gb|CN749026.1|CN749026 | / | | / | | No hit | |  |  |  | 1.7 |
| ApAL3SD-III-C6 | CL190Contig2 | / | | / | | No hit | |  |  | -1.9 | -1.5 |
| ApAL3SD-III-D7 | gi|47514080|gb|CN749083.1|CN749083 | / | | / | | No hit | |  |  |  | 1.4 |
| ApAL3SD-III-E6 | CL1433Contig1 | / | | / | | No hit | |  |  | 1.3 | 1.3 |
| ApAL3SD-III-F6 | CL3419Contig1 | / | | / | | No hit | |  |  | 1.6 | -1.8 |
| ApAL3SD-IV-A5 | CL1178Contig1 | / | | / | | No hit | |  |  |  | -2.2 |
| ApAL3SD-IV-C1 | CL122Contig1 | / | | / | | No hit | |  |  |  | -1.9 |
| ApAL3SD-IV-C5 | gi|47514126|gb|CN749129.1|CN749129 | / | | / | | No hit | |  |  |  | -1.3 |
| ApAL3SD-IV-G3 | CL1394Contig1 | / | | / | | No hit | |  |  | -1.4 |  |
| ApAL3SD-V-A4 | gi|47514167|gb|CN749170.1|CN749170 | / | | / | | No hit | |  |  |  | 1.3 |
| ApAL3SD-V-E9 | gi|47514202|gb|CN749205.1|CN749205 | / | | / | | No hit | |  |  | -3.6 | -2.5 |
| ApAL3SD-VI-B4 | gi|47514232|gb|CN749235.1|CN749235 | / | | / | | No hit | |  |  |  | 2.0 |
| ApAL3SD-VI-F3 | CL1Contig167 | / | | / | | No hit | |  |  | 1.3 |  |
| ApAL3SD-VIII-H8 | CL2072Contig1 | / | | / | | No hit | |  |  |  | 1.9 |
| ApAL3SD-XII-B1 | gi|47514463|gb|CN749466.1|CN749466 | / | | / | | No hit | |  |  | -1.6 |  |
| ApAL3SD-XII-E9 | gi|47514486|gb|CN749489.1|CN749489 | / | | / | | No hit | |  |  | -1.7 |  |
| ApAL3SD-XII-G7 | gi|47514500|gb|CN749503.1|CN749503 | / | | / | | No hit | |  |  | -1.6 | -1.6 |
| ApAL3SD-XIII-G11 | CL2037Contig2 | / | | / | | No hit | |  |  |  | 1.6 |
| ApAL3SD-XIV-A6 | CL10973Contig1 | / | | / | | No hit | |  |  |  | 1.8 |
| ApAL3SD-XIV-C5 | gi|47514573|gb|CN749576.1|CN749576 | / | | / | | No hit | |  |  |  | 1.9 |
| ApAL3SD-XIV-E4 | CL11589Contig1 | / | | / | | No hit | |  |  | -1.3 | -1.4 |
| ApAL3SD-XIV-G12 | CL1793Contig2 | / | | / | | No hit | |  |  | 1.3 |  |
| ApAL3SD-XIX-C3 | CL9253Contig1 | / | | / | | No hit | |  |  | 1.5 | 1.6 |
| ApAL3SD-XIX-D9 | CL3406Contig1 | / | | / | | No hit | |  |  |  | 1.4 |
| ApAL3SD-XIX-G11 | gi|47514643|gb|CN749646.1|CN749646 | / | | / | | No hit | |  |  |  | -1.5 |
| ApAL3SD-XIX-G5 | gi|47514646|gb|CN749649.1|CN749649 | / | | / | | No hit | |  |  | -1.3 |  |
| ApAL3SD-XV-D8 | CL6912Contig1 | / | | / | | No hit | |  |  | -1.3 | -1.5 |
| ApAL3SD-XVI-A4 | gi|47514702|gb|CN749705.1|CN749705 | / | | / | | No hit | |  |  | -1.3 | -1.9 |
| ApAL3SD-XVII-A8 | gi|47514759|gb|CN749762.1|CN749762 | / | | / | | No hit | |  |  | -1.3 | -1.6 |
| ApAL3SD-XVII-B11 | CL8730Contig1 | / | | / | | No hit | |  |  | -1.7 | -2.3 |
| ApAL3SD-XVII-D2 | gi|47514777|gb|CN749780.1|CN749780 | / | | / | | No hit | |  |  |  | -1.7 |
| ApAL3SD-XVII-E7 | CL2623Contig1 | / | | / | | No hit | |  |  |  | 1.3 |
| ApAL3SD-XVIII-G3 | gi|47514851|gb|CN749854.1|CN749854 | / | | / | | No hit | |  |  | -1.3 |  |
| ApAL3SD-XX-F10 | CL12346Contig1 | / | | / | | No hit | |  |  |  | -1.6 |
| ApDT-XVIII-F7 | CL8098Contig1 | / | | / | | No hit | |  |  |  | 1.3 |
| ApDT-XXVI-A3 | CL1Contig235 | / | | / | | No hit | |  |  | 1.3 |  |
| ApHL3LD-III-A6 | gi|47517518|gb|CN752521.1|CN752521 | / | | / | | No hit | | 1.2 |  |  | 2.2 |
| ApHL3LD-III-D11 | CL1Contig353 | / | | / | | No hit | |  |  |  | 1.4 |
| ApHL3LD-III-D8 | gi|47517547|gb|CN752550.1|CN752550 | / | | / | | No hit | |  |  |  | -1.5 |
| ApHL3LD-IV-A2 | CL3911Contig1 | / | | / | | No hit | |  |  | 1.3 | 1.4 |
| ApHL3LD-IV-H2 | gi|47517654|gb|CN752657.1|CN752657 | / | | / | | No hit | |  |  |  | 1.6 |
| ApHL3LD-X-D6 | CL1Contig511 | / | | / | | No hit | |  |  | 1.3 | 1.5 |
| ApHL3LD-X-G12 | CL1678Contig1 | / | | / | | No hit | |  |  |  | -1.6 |
| ApHL3LD-XI-C10 | CL939Contig1 | / | | / | | No hit | |  |  | 1.4 | 2.9 |
| ApHL3LD-XI-C3 | gi|47518028|gb|CN753031.1|CN753031 | / | | / | | No hit | |  |  | 1.4 | 1.4 |
| ApHL3LD-XIII-D2 | gi|47518099|gb|CN753102.1|CN753102 | / | | / | | No hit | |  |  | 1.3 | 1.4 |
| ApHL3LD-XIV-C12 | gi|47518144|gb|CN753147.1|CN753147 | / | | / | | No hit | |  |  | 1.3 | 1.6 |
| ApHL3LD-XVII-B5 | CL9464Contig1 | / | | / | | No hit | |  | 1.4 | -3.3 | -2.9 |
| ApHL3LD-XVII-D11 | CL8375Contig1 | / | | / | | No hit | |  |  | 1.5 | 1.4 |
| ApHL3LD-XVII-G12 | gi|47518295|gb|CN753298.1|CN753298 | / | | / | | No hit | |  |  |  | 2.5 |
| ApHL3LD-XVIII-G2 | gi|47518353|gb|CN753356.1|CN753356 | / | | / | | No hit | |  |  |  | 1.6 |
| ApHL3LD-XVIII-H2 | gi|47518359|gb|CN753362.1|CN753362 | / | | / | | No hit | |  |  |  | 1.7 |
| ApHL3SD-I-C6 | gi|47516035|gb|CN751038.1|CN751038 | / | | / | | No hit | |  |  | -1.4 | -1.5 |
| ApHL3SD-III-A1 | gi|47516149|gb|CN751152.1|CN751152 | / | | / | | No hit | |  |  | 1.3 | 1.5 |
| ApHL3SD-III-E12 | gi|47516181|gb|CN751184.1|CN751184 | / | | / | | No hit | |  |  |  | 1.4 |
| ApHL3SD-III-F9 | CL1089Contig1 | / | | / | | No hit | |  |  | 3.5 |  |
| ApHL3SD-IV-F4 | gi|47516259|gb|CN751262.1|CN751262 | / | | / | | No hit | |  |  | 1.3 | 2.7 |
| ApHL3SD-V-B1 | CL10627Contig1 | / | | / | | No hit | |  |  | -1.9 |  |
| ApHL3SD-V-B7 | gi|47516300|gb|CN751303.1|CN751303 | / | | / | | No hit | |  |  | -1.3 | -1.5 |
| ApHL3SD-V-C5 | gi|47516306|gb|CN751309.1|CN751309 | / | | / | | No hit | |  |  | -2.0 | -1.2 |
| ApHL3SD-VI-A7 | CL5189Contig1 | / | | / | | No hit | |  |  | -1.3 | -1.6 |
| ApHL3SD-VI-A8 | gi|47516362|gb|CN751365.1|CN751365 | / | | / | | No hit | |  |  | -1.4 | -1.5 |
| ApHL3SD-VII-C4 | CL41Contig2 | / | | / | | No hit | |  |  |  | 1.4 |
| ApHL3SD-XII-B1 | gi|47516544|gb|CN751547.1|CN751547 | / | | / | | No hit | |  |  |  | 1.9 |
| ApHL3SD-XII-D4 | gi|47516559|gb|CN751562.1|CN751562 | / | | / | | No hit | |  |  | 1.3 | 1.5 |
| ApHL3SD-XIII-C6 | gi|47516595|gb|CN751598.1|CN751598 | / | | / | | No hit | |  |  | -2.3 | -2.8 |
| ApHL3SD-XIII-C7 | gi|47516596|gb|CN751599.1|CN751599 | / | | / | | No hit | | -1.9 |  | -4.1 | -18.5 |
| ApHL3SD-XIII-D5 | gi|47516602|gb|CN751605.1|CN751605 | / | | / | | No hit | |  |  |  | 1.5 |
| ApHL3SD-XIII-F2 | gi|47516615|gb|CN751618.1|CN751618 | / | | / | | No hit | |  |  | 1.3 | 1.4 |
| ApHL3SD-XXIX-E2 | gi|47517061|gb|CN752064.1|CN752064 | / | | / | | No hit | |  |  | 1.3 | 1.5 |
| ApHL3SD-XXV-F12 | gi|47517134|gb|CN752137.1|CN752137 | / | | / | | No hit | |  |  |  | 3.7 |
| ApHL3SD-XXVII-B8 | gi|47517175|gb|CN752178.1|CN752178 | / | | / | | No hit | |  |  | -1.4 |  |
| ApHL3SD-XXVII-G1 | gi|47517210|gb|CN752213.1|CN752213 | / | | / | | No hit | |  |  |  | 2.6 |
| ApHL3SD-XXX-F2 | gi|47517315|gb|CN752318.1|CN752318 | / | | / | | No hit | |  |  |  | 3.5 |
| ApHL3SD-XXX-F4 | CL8050Contig1 | / | | / | | No hit | |  |  |  | 1.5 |
| ApHL3SD-XXXI-B3 | CL2034Contig1 | / | | / | | No hit | |  |  | -1.8 | -1.9 |
| ApHL3SD-XXXII-D7 | gi|47517400|gb|CN752403.1|CN752403 | / | | / | | No hit | |  |  | 1.3 | 1.3 |
| ID0AAG11CA10CM1 | gi|82572206|gb|DV743862.1|DV743862 | / | | / | | No hit | |  |  | 1.2 | 1.4 |
| ID0AAG11CE07CM1 | gi|82572238|gb|DV743894.1|DV743894 | / | | / | | No hit | |  |  | 1.4 | 1.4 |
| ID0AAG12AE12CM1 | CL1Contig832 | / | | / | | No hit | |  |  | 1.3 | 1.4 |
| ID0AAG12DA09CM1 | gi|82572537|gb|DV744193.1|DV744193 | / | | / | | No hit | |  |  | 1.4 | 1.5 |
| ID0AAG1CC03CM1 | gi|82573546|gb|DV745202.1|DV745202 | / | | / | | No hit | |  |  | 1.4 | 1.5 |
| ID0AAG2CF08CM1 | gi|82573837|gb|DV745493.1|DV745493 | / | | / | | No hit | |  |  | 1.4 | 1.6 |
| ID0AAG3AD01CM1 | gi|82573957|gb|DV745613.1|DV745613 | / | | / | | No hit | |  |  | 1.3 | 1.4 |
| ID0AAG5AG08CM1 | gi|82574555|gb|DV746211.1|DV746211 | / | | / | | No hit | |  |  | 1.4 | 1.4 |
| ID0AAH13BF06ZM2 | CL4920Contig1 | / | | / | | No hit | |  |  |  | -1.3 |
| ID0AAH15BB08ZM1 | CL3722Contig1 | / | | / | | No hit | |  |  |  | -1.4 |
| ID0AAH2CF02ZM1 | gi|82578164|gb|DV749820.1|DV749820 | / | | / | | No hit | |  |  |  | -1.3 |
| ID0AAH3BG11ZM1 | gi|82578401|gb|DV750057.1|DV750057 | / | | / | | No hit | |  |  |  | -1.4 |
| ID0AAH4AC05ZM1 | CL93Contig1 | / | | / | | No hit | |  |  |  | -1.4 |
| ID0AAH5CE11ZM1 | gi|82579043|gb|DV750699.1|DV750699 | / | | / | | No hit | |  |  |  | -1.4 |
| ID0AAH6CC06ZM1 | gi|82579342|gb|DV750998.1|DV750998 | / | | / | | No hit | |  |  |  | 1.3 |
| ID0AAH6CD12ZM1 | gi|82579356|gb|DV751012.1|DV751012 | / | | / | | No hit | |  |  |  | 1.3 |
| ID0AAH7BA11ZM1 | gi|82579549|gb|DV751205.1|DV751205 | / | | / | | No hit | |  |  | 1.4 | 1.7 |
| ID0AAH7CA06ZM1 | gi|82579624|gb|DV751280.1|DV751280 | / | | / | | No hit | |  |  |  | -1.4 |
| ID0AAH7CA12ZM1 | CL6206Contig1 | / | | / | | No hit | |  |  |  | 1.5 |
| ID0AAH7CB06ZM1 | CL9684Contig1 | / | | / | | No hit | |  |  |  | -1.3 |
| ID0AAH7CC11ZM1 | gi|82579648|gb|DV751304.1|DV751304 | / | | / | | No hit | |  |  | 1.8 | 2.3 |
| ID0AAH7DD02ZM1 | gi|82579724|gb|DV751380.1|DV751380 | / | | / | | No hit | |  |  |  | -1.5 |
| ID0AAH7DH11ZM1 | CL3061Contig1 | / | | / | | No hit | |  |  | 1.3 | 1.4 |
| ID0AAH8CH01ZM1 | gi|82579970|gb|DV751626.1|DV751626 | / | | / | | No hit | |  |  |  | -1.4 |
| ID0AAK10YA22CM1 | CL8021Contig1 | / | | / | | No hit | |  |  |  | -1.4 |
| ID0AAK10YC06CM1 | gi|86461208|gb|DY227080.1|DY227080 | / | | / | | No hit | |  |  |  | -1.3 |
| ID0AAK10YC19CM1 | CL768Contig1 | / | | / | | No hit | |  |  | 2.2 | -1.8 |
| ID0AAK10YD11CM1 | gi|86461237|gb|DY227109.1|DY227109 | / | | / | | No hit | |  |  | 1.3 |  |
| ID0AAK10YF18CM1 | CL159Contig2 | / | | / | | No hit | |  |  |  | 1.4 |
| ID0AAK10YH02CM1 | gi|86461319|gb|DY227191.1|DY227191 | / | | / | | No hit | |  |  | -5.0 |  |
| ID0AAK10YK24CM1 | CL3103Contig1 | / | | / | | No hit | |  |  |  | 1.5 |
| ID0AAK10YL05CM1 | CL11031Contig1 | / | | / | | No hit | |  |  |  | 1.3 |
| ID0AAK10YN04CM1 | CL7871Contig1 | / | | / | | No hit | |  |  | 4.1 |  |
| ID0AAK1YB05CM1 | CL7498Contig1 | / | | / | | No hit | |  |  |  | 1.5 |
| ID0AAK1YC01CM1 | gi|86461566|gb|DY227438.1|DY227438 | / | | / | | No hit | |  | 2.1 | -5.1 | 2.6 |
| ID0AAK1YC03CM1 | CL1Contig1283 | / | | / | | No hit | |  |  | 1.3 |  |
| ID0AAK1YD17CM1 | CL5720Contig1 | / | | / | | No hit | |  |  |  | 1.5 |
| ID0AAK1YE11CM1 | CL416Contig1 | / | | / | | No hit | |  |  | 1.6 | 1.4 |
| ID0AAK1YE20CM1 | gi|86461627|gb|DY227499.1|DY227499 | / | | / | | No hit | |  |  | -1.6 | -1.7 |
| ID0AAK1YJ02CM1 | gi|86461717|gb|DY227589.1|DY227589 | / | | / | | No hit | |  |  | -1.3 | -1.5 |
| ID0AAK1YJ06CM1 | CL1Contig14 | / | | / | | No hit | |  |  |  | -1.3 |
| ID0AAK1YL04CM1 | gi|86461759|gb|DY227631.1|DY227631 | / | | / | | No hit | |  |  | -1.8 | -2.7 |
| ID0AAK1YL18CM1 | gi|86461771|gb|DY227643.1|DY227643 | / | | / | | No hit | |  |  | 1.5 |  |
| ID0AAK1YL24CM1 | gi|86461774|gb|DY227646.1|DY227646 | / | | / | | No hit | |  |  | 2.0 | 3.3 |
| ID0AAK1YM07CM1 | gi|86461780|gb|DY227652.1|DY227652 | / | | / | | No hit | |  |  |  | 1.9 |
| ID0AAK1YM20CM1 | CL1Contig1390 | / | | / | | No hit | |  |  |  | 9.4 |
| ID0AAK1YP06CM1 | gi|86461841|gb|DY227713.1|DY227713 | / | | / | | No hit | |  |  |  | 1.3 |
| ID0AAK1YP16CM1 | gi|86461851|gb|DY227723.1|DY227723 | / | | / | | No hit | |  |  |  | 1.5 |
| ID0AAK2YD22CM1 | gi|86461939|gb|DY227811.1|DY227811 | / | | / | | No hit | |  |  | -3.9 | -9.9 |
| ID0AAK2YD23CM1 | gi|86461940|gb|DY227812.1|DY227812 | / | | / | | No hit | |  |  | 2.4 | -4.9 |
| ID0AAK2YF24CM1 | gi|86461983|gb|DY227855.1|DY227855 | / | | / | | No hit | |  |  | 1.3 | 1.6 |
| ID0AAK2YG14CM1 | CL127Contig1 | / | | / | | No hit | | -2.3 |  | -5.1 | -22.8 |
| ID0AAK2YG19CM1 | gi|86462000|gb|DY227872.1|DY227872 | / | | / | | No hit | | -1.5 |  | -4.1 | -3.3 |
| ID0AAK2YL07CM1 | gi|86462094|gb|DY227966.1|DY227966 | / | | / | | No hit | |  |  |  | 2.2 |
| ID0AAK2YN21CM1 | gi|86462148|gb|DY228020.1|DY228020 | / | | / | | No hit | |  |  |  | 1.5 |
| ID0AAK2YP02CM1 | gi|86462176|gb|DY228048.1|DY228048 | / | | / | | No hit | |  |  | 1.5 | 1.7 |
| ID0AAK3YD03CM1 | gi|86462259|gb|DY228131.1|DY228131 | / | | / | | No hit | | -2.4 |  | -7.1 |  |
| ID0AAK3YE18CM1 | CL11118Contig1 | / | | / | | No hit | |  |  |  | -1.5 |
| ID0AAK3YI12CM1 | gi|86462371|gb|DY228243.1|DY228243 | / | | / | | No hit | |  |  | 1.5 | 1.6 |
| ID0AAK3YL18CM1 | CL85Contig1 | / | | / | | No hit | |  |  | 10.4 | 55.8 |
| ID0AAK3YM11CM1 | CL11223Contig1 | / | | / | | No hit | |  |  | 1.5 |  |
| ID0AAK3YO01CM1 | CL1Contig208 | / | | / | | No hit | |  |  | -3.4 | 5.0 |
| ID0AAK3YO04CM1 | gi|86462489|gb|DY228361.1|DY228361 | / | | / | | No hit | |  |  | -2.4 | 3.4 |
| ID0AAK3YO14CM1 | gi|86462498|gb|DY228370.1|DY228370 | / | | / | | No hit | |  |  |  | 6.7 |
| ID0AAK4YB08CM1 | gi|86462557|gb|DY228429.1|DY228429 | / | | / | | No hit | |  |  | -3.9 |  |
| ID0AAK4YB12CM1 | CL1Contig907 | / | | / | | No hit | |  |  | -5.0 |  |
| ID0AAK4YD14CM1 | gi|86462607|gb|DY228479.1|DY228479 | / | | / | | No hit | |  |  | 2.2 | 47.3 |
| ID0AAK4YD18CM1 | gi|86462611|gb|DY228483.1|DY228483 | / | | / | | No hit | |  |  |  | 1.5 |
| ID0AAK4YE22CM1 | CL1235Contig1 | / | | / | | No hit | |  |  | -1.9 |  |
| ID0AAK4YG18CM1 | gi|86462672|gb|DY228544.1|DY228544 | / | | / | | No hit | |  |  |  | 1.3 |
| ID0AAK4YG24CM1 | CL8195Contig1 | / | | / | | No hit | |  |  | -1.3 | 1.6 |
| ID0AAK4YI01CM1 | CL815Contig2 | / | | / | | No hit | |  |  |  | 2.2 |
| ID0AAK4YI20CM1 | gi|86462717|gb|DY228589.1|DY228589 | / | | / | | No hit | |  |  | -1.3 |  |
| ID0AAK4YK12CM1 | gi|86462753|gb|DY228625.1|DY228625 | / | | / | | No hit | | -1.4 |  | -1.9 | -3.7 |
| ID0AAK4YL04CM1 | gi|86462768|gb|DY228640.1|DY228640 | / | | / | | No hit | |  | 1.8 | -3.0 | 2.2 |
| ID0AAK4YM06CM1 | CL5106Contig1 | / | | / | | No hit | |  | 3.3 |  | -8.0 |
| ID0AAK4YM08CM1 | gi|86462792|gb|DY228664.1|DY228664 | / | | / | | No hit | |  |  | 1.4 | 6.1 |
| ID0AAK4YO15CM1 | gi|86462838|gb|DY228710.1|DY228710 | / | | / | | No hit | |  |  | -8.1 | -7.0 |
| ID0AAK5YB04CM1 | gi|86462886|gb|DY228758.1|DY228758 | / | | / | | No hit | |  |  | -2.3 | 3.3 |
| ID0AAK5YI19CM1 | gi|86463047|gb|DY228919.1|DY228919 | / | | / | | No hit | | -1.5 |  | -1.5 | -3.7 |
| ID0AAK5YI20CM1 | gi|86463048|gb|DY228920.1|DY228920 | / | | / | | No hit | |  |  |  | -1.5 |
| ID0AAK5YJ03CM1 | CL1Contig628 | / | | / | | No hit | |  |  | 1.5 | 1.6 |
| ID0AAK5YN03CM1 | CL8177Contig1 | / | | / | | No hit | |  |  |  | -1.3 |
| ID0AAK5YN10CM1 | gi|86463135|gb|DY229007.1|DY229007 | / | | / | | No hit | |  |  |  | 6.3 |
| ID0AAK5YP15CM1 | CL10734Contig1 | / | | / | | No hit | |  |  | -1.3 | -2.0 |
| ID0AAK6YG19CM1 | gi|86463349|gb|DY229221.1|DY229221 | / | | / | | No hit | |  |  | 1.6 | 1.8 |
| ID0AAK6YH16CM1 | CL2354Contig2 | / | | / | | No hit | |  |  |  | -1.7 |
| ID0AAK6YH18CM1 | CL1Contig1226 | / | | / | | No hit | |  |  |  | 9.1 |
| ID0AAK6YI05CM1 | gi|86463381|gb|DY229253.1|DY229253 | / | | / | | No hit | |  |  |  | 1.5 |
| ID0AAK6YI09CM1 | CL2090Contig1 | / | | / | | No hit | |  |  | -2.2 | -3.4 |
| ID0AAK6YJ08CM1 | gi|86463407|gb|DY229279.1|DY229279 | / | | / | | No hit | |  |  |  | 1.4 |
| ID0AAK6YK06CM1 | gi|86463427|gb|DY229299.1|DY229299 | / | | / | | No hit | |  |  | 2.1 | 2.8 |
| ID0AAK6YK20CM1 | CL1676Contig1 | / | | / | | No hit | |  |  |  | 1.6 |
| ID0AAK6YK21CM1 | gi|86463442|gb|DY229314.1|DY229314 | / | | / | | No hit | |  |  | -1.7 | 2.9 |
| ID0AAK6YN24CM1 | gi|86463511|gb|DY229383.1|DY229383 | / | | / | | No hit | |  |  |  | 1.6 |
| ID0AAK7YE10CM1 | CL1Contig320 | / | | / | | No hit | |  |  | 2.3 | -1.4 |
| ID0AAK7YI19CM1 | CL284Contig2 | / | | / | | No hit | |  |  | 1.6 | 1.7 |
| ID0AAK7YN20CM1 | CL9403Contig1 | / | | / | | No hit | |  |  | -1.5 | 1.8 |
| ID0AAK8YH01CM1 | gi|86464047|gb|DY229919.1|DY229919 | / | | / | | No hit | |  |  | -2.1 |  |
| ID0AAK8YK20CM1 | CL972Contig1 | / | | / | | No hit | |  |  | -1.2 | 1.4 |
| ID0AAK9YC08CM1 | CL2780Contig1 | / | | / | | No hit | |  |  |  | -2.0 |
| ID0AAK9YC15CM1 | gi|86464287|gb|DY230159.1|DY230159 | / | | / | | No hit | |  |  |  | 1.4 |
| ID0AAK9YD13CM1 | CL1Contig522 | / | | / | | No hit | |  |  | 1.3 |  |
| ID0AAK9YD19CM1 | gi|86464310|gb|DY230182.1|DY230182 | / | | / | | No hit | |  |  | 1.4 |  |
| ID0AAK9YD23CM1 | gi|86464314|gb|DY230186.1|DY230186 | / | | / | | No hit | |  |  |  | 1.4 |
| ID0AAK9YE02CM1 | gi|86464317|gb|DY230189.1|DY230189 | / | | / | | No hit | |  |  | 1.4 |  |
| ID0AAK9YF09CM1 | gi|86464344|gb|DY230216.1|DY230216 | / | | / | | No hit | |  |  | -1.5 |  |
| ID0AAK9YI07CM1 | CL984Contig1 | / | | / | | No hit | |  |  |  | 2.5 |
| ID0AAK9YO04CM1 | gi|86464534|gb|DY230406.1|DY230406 | / | | / | | No hit | |  |  | 1.6 | 2.2 |
| ID0AFF11DA06CM1 | gi|82563123|gb|DV738813.1|DV738813 | / | | / | | No hit | |  |  | 1.3 |  |
| ID0AFF12DB01CM1 | gi|82563476|gb|DV739166.1|DV739166 | / | | / | | No hit | |  |  | 1.3 |  |
| ID0AFF13AH08CM1 | CL511Contig2 | / | | / | | No hit | |  |  | 1.3 | 1.4 |
| ID0AFF15CE10CM1 | gi|82564482|gb|DV740172.1|DV740172 | / | | / | | No hit | |  |  | 1.3 |  |
| ID0AFF1BC11CM1 | gi|82564713|gb|DV740403.1|DV740403 | / | | / | | No hit | |  |  | 1.4 | 1.4 |
| ID0AFF2AA04CM1 | gi|82564939|gb|DV740629.1|DV740629 | / | | / | | No hit | |  |  | 1.3 |  |
| ID0AFF2AE11CM1 | gi|82564991|gb|DV740681.1|DV740681 | / | | / | | No hit | |  |  | 1.3 |  |
| ID0AFF3AA06CM1 | CL4250Contig1 | / | | / | | No hit | |  |  |  | 1.3 |
| ID0AFF3BG04CM1 | CL2069Contig2 | / | | / | | No hit | |  |  | 1.3 | 1.4 |
| ID0AFF3CD12CM1 | gi|82565502|gb|DV741192.1|DV741192 | / | | / | | No hit | |  |  | 1.4 |  |
| ID0AFF3CE04CM1 | gi|82565506|gb|DV741196.1|DV741196 | / | | / | | No hit | |  |  | 1.4 | 1.6 |
| ID0AFF3CH03CM1 | gi|82565539|gb|DV741229.1|DV741229 | / | | / | | No hit | |  |  | 1.3 |  |
| ID0AFF5AA06CM1 | gi|82566001|gb|DV741691.1|DV741691 | / | | / | | No hit | |  |  |  | 1.4 |
| ID0AFF6DC11CM1 | CL1Contig1190 | / | | / | | No hit | |  |  | 1.3 |  |
| ID0AFF6DG05CM1 | CL2431Contig1 | / | | / | | No hit | |  |  | 1.3 |  |
| ID0AFF7CC09CM1 | CL9402Contig1 | / | | / | | No hit | |  |  | -1.5 | -1.3 |
| ID0AFF7CC10CM1 | CL1Contig768 | / | | / | | No hit | |  |  | -1.6 | -1.3 |
| ID0AFF7DE07CM1 | gi|82567021|gb|DV742711.1|DV742711 | / | | / | | No hit | |  |  |  | 1.3 |
| ID0AFF7DF07CM1 | gi|82567033|gb|DV742723.1|DV742723 | / | | / | | No hit | |  |  | 1.3 | 1.5 |
| ID0AFF7DH01CM1 | gi|82567049|gb|DV742739.1|DV742739 | / | | / | | No hit | |  |  |  | 1.4 |
| ID0AFF9AH03CM1 | CL683Contig1 | / | | / | | No hit | |  |  |  | -1.4 |
| Ap_SDD1_1D06_T7 | CL_AP_13684.5-ID0AEE9BE10RM2 | / | | / | | No Hit | |  |  | -1.7 |  |
| Ap_SDD1_1G11_T7 | No Contig | / | | / | | No Hit | |  |  | 1.5 |  |
| Ap_SDD1_5E05_SP6 | CTG_AP_380.4-ID0AAA28AB12RM1 | / | | / | | No Hit | |  |  | 1.5 |  |
| Ap_SDD3_1H02_SP6 | CL_AP_12257.5-ID0AAI9YL03RM1 | / | | / | | No Hit | |  |  |  | -1.7 |
| Ap_SDD3_2E01_SP6 | No Contig | / | | / | | No Hit | |  |  | 1.7 |  |
| Ap_SDD3_2G12_SP6 | No Contig | / | | / | | No Hit | |  |  | 1.8 |  |
| Ap_SDU2_2H02_SP6 | No Contig | / | | / | | No Hit | |  |  | 2.9 |  |
| Ap_SDU2_3D12_SP6 | No Contig | / | | / | | No Hit | |  |  | -2.2 | -3.3 |
| Ap_SDU2_3H10_SP6 | No Contig | / | | / | | No Hit | |  |  | 2.7 |  |
| MpW-II-H07 | CL_MP_4790.3-34966101 | / | | / | | No Hit | |  |  |  | -1.3 |
| MpW-III-E05 | CL_MP_5788.3-34966070 | / | | / | | No Hit | |  |  | -1.9 | -1.5 |
| MpW-IV-A08 | CL_MP_6696.3-34966238 | / | | / | | No Hit | |  |  | 1.4 |  |
| MpW-IV-C11 | CTG_MP_2522.3-34966001 | / | | / | | No Hit | |  |  | 1.3 | 1.5 |
| MpW-IV-D05 | CL_MP_7200.3-34966307 | / | | / | | No Hit | |  |  | -1.6 |  |
| MpW-V-A02 | CL_MP_6763.3-34966130 | / | | / | | No Hit | |  |  | 1.3 |  |
| MpW-VI-D03 | CL_MP_7032.3-34966340 | / | | / | | No Hit | | 1.3 |  |  | 2.2 |
| MpW-VI-E01 | CL_MP_4891.3-34966008 | / | | / | | No Hit | |  |  | 1.5 |  |
| MpW-VII-B07 | CL_MP_4635.3-34966251 | / | | / | | No Hit | |  |  | -2.6 |  |
| MpW-VII-G07 | CL_MP_5353.3-34966185 | / | | / | | No Hit | |  |  | 1.4 | 1.3 |
| MpW-VIII-H02 | CL_MP_7702.3-34966428 | / | | / | | No Hit | | -1.9 |  | -3.1 | -7.0 |
| MpW-X-B09 | CL_MP_5139.3-34966355 | / | | / | | No Hit | | -1.5 |  | -1.9 | -4.1 |
| MpW-X-B10 | CL_MP_7274.3-34966369 | / | | / | | No Hit | |  |  | -2.0 |  |
| MpW-X-D03 | CL_MP_8894.3-34966414 | / | | / | | No Hit | |  |  |  | 2.0 |
| MpW-XII-B04 | CL_MP_4338.3-34966338 | / | | / | | No Hit | |  |  |  | 4.4 |
| MpW-XII-G03 | CL_MP_4832.3-34966625 | / | | / | | No Hit | |  |  |  | 2.0 |
| MpW-XII-H05 | CL_MP_7166.3-34966680 | / | | / | | No Hit | |  |  | 1.3 |  |
| MpW-XIV-H03 | CL_MP_4333.3-34966564 | / | | / | | No Hit | |  |  | 1.8 | 1.5 |
| MpW-XV-A04 | CL_MP_8003.3-34966690 | / | | / | | No Hit | |  | 1.6 | -4.6 |  |
| MpW-XV-A05 | CTG_MP_419.3-34597730 | / | | / | | No Hit | |  |  |  | 8.4 |
| MpW-XV-A06 | CL_MP_8228.3-34966585 | / | | / | | No Hit | |  |  | 1.3 |  |
| MpW-XV-C11 | CTG_MP_1168.3-34966350 | / | | / | | No Hit | |  |  |  | 1.7 |
| MpW-XV-D06 | CTG_MP_419.3-34597730 | / | | / | | No Hit | |  |  | 1.5 | 5.1 |
| MpW-XV-E12 | CL_MP_6690.3-34966768 | / | | / | | No Hit | | -3.0 |  | -3.6 | -13.4 |
| MpW-XVIII-G07 | CL_MP_8852.3-34966888 | / | | / | | No Hit | |  | 1.6 | -2.3 | 1.5 |
| ID0AAK4YI02CM1 | CL41Contig1 | A0BFH1 | | 3.00E-25 | | Hypothetical protein | |  |  | 4.7 | 10.3 |
| ID0AAK4YL07CM1 |  | A0BFH1 | | 3.00E-25 | | Hypothetical protein | |  |  | 4.7 | 9.4 |
| ID0AAK5YD16CM1 |  | A0BFH1 | | 3.00E-25 | | Hypothetical protein | |  |  | 2.8 | 6.8 |
| ID0AAK5YP10CM1 |  | A0BFH1 | | 3.00E-25 | | Hypothetical protein | |  |  | 4.2 | 7.2 |
| ApHL3SD-XXXIII-G5 | CL1Contig1229 | B1U3M9 | | 1.00E-11 | | Hypothetical protein | |  |  | -4.6 |  |
| ID0AAK1YG08CM1 |  | B1U3M9 | | 1.00E-11 | | Hypothetical protein | |  |  | -4.1 |  |
| ID0AAK4YP12CM1 |  | B1U3M9 | | 1.00E-11 | | Hypothetical protein | |  |  | -5.5 |  |
| ID0AAK8YC09CM1 |  | B1U3M9 | | 1.00E-11 | | Hypothetical protein | |  |  | -1.4 |  |
| ID0AAK1YC20CM1 | CL6318Contig1 | Q2JFA5 | | 3E-15 | | Hypothetical protein | |  |  | -1.8 | -3.2 |
| ApAL3SD-XI-G6 | CL3547Contig1 | Q29B63 | | 1.00E-107 | | Hypothetical protein | |  |  | -1.3 |  |
| ApAL3SD-XIV-A2 | CL1Contig634 | Q0E906 | | 7E-14 | | Hypothetical protein | |  |  | 1.5 |  |
| ApAL3SD-XVIII-D1 | CL5589Contig1 | Q2LYS2 | | 7.00E-23 | | Hypothetical protein | |  |  | -1.3 | -1.7 |
| ApDT-XX-H5 | CL2438Contig1 | Q59JR6 | | 1.00E-37 | | Hypothetical protein | |  |  | -1.3 |  |
| ApHL3LD-III-H6 | CL4788Contig1 | Q9W3C2 | | 2.00E-12 | | Hypothetical protein | |  |  |  | 2.7 |
| ApHL3LD-IV-G8 | CL635Contig1 | Q9VN73 | | 4.00E-57 | | Hypothetical protein | |  |  | -3.5 | -3.5 |
| ApHL3LD-VIII-C2 | CL1Contig1331 | Q57T06 | | 7.00E-38 | | Hypothetical protein | |  |  | -2.8 |  |
| ApHL3LD-XVII-B8 | gi|47531672|gb|CN757749.1|CN757749 | Q9VHC5 | | 2.00E-31 | | Hypothetical protein | |  |  | -1.4 | -1.3 |
| ApHL3LD-XVII-G11 | CL160Contig2 | Q9VRM5 | | 1.00E-34 | | Hypothetical protein | |  |  | 1.3 | 1.6 |
| ApHL3SD-I-D7 | CL3871Contig1 | Q4S0Q2 | | 3.00E-29 | | Hypothetical protein | |  |  | -1.3 | -1.4 |
| ApHL3SD-II-D11 | CL1Contig1259 | Q5C0F6 | | 5E-18 | | Hypothetical protein | |  |  |  | 2.9 |
| ApHL3SD-IV-C5 | CL2368Contig1 | Q86BG3 | | 4.00E-06 | | Hypothetical protein | |  |  | -1.5 |  |
| ApHL3SD-V-D9 | CL1554Contig1 | Q7QIK3 | | 6.00E-46 | | Hypothetical protein | |  |  |  | 2.1 |
| ApHL3SD-XII-E8 | CL1Contig530 | Q2PDX2 | | 1E-14 | | Hypothetical protein | |  |  | -1.6 | 1.5 |
| ApHL3SD-XVII-A12 | CL1Contig666 | Q9VFV1 | | 1.00E-85 | | Hypothetical protein | | -1.2 |  | -1.5 | -3.1 |
| ApHL3SD-XXIV-B1 | gi|47516982|gb|CN751985.1|CN751985 | A2AJT9 | | 4.00E-11 | | Hypothetical protein | |  |  | -3.0 | -5.0 |
| ApHL3SD-XXIX-C7 | gi|86458932|gb|DY224804.1|DY224804 | Q09JM0 | | 1E-13 | | Hypothetical protein | |  |  |  | 2.5 |
| ID0AAH11AC08ZM1 | CL2396Contig1 | Q9VN71 | | 5.00E-29 | | Hypothetical protein | |  |  |  | -1.3 |
| ID0AAH2BB08ZM1 | CL309Contig3 | Q9VPS3 | | 2.00E-07 | | Hypothetical protein | |  |  |  | -1.7 |
| ID0AAH5CE10ZM1 | CL9862Contig1 | Q9VN71 | | 1.00E-05 | | Hypothetical protein | |  |  |  | -4.2 |
| ID0AAH6BA10ZM1 | CL1451Contig1 | Q9VVW7 | | 2.00E-59 | | Hypothetical protein | |  |  |  | -4.2 |
| ID0AAH7BG10ZM1 | CL2196Contig1 | Q8SXE9 | | 5.00E-24 | | Hypothetical protein | |  |  |  | -1.5 |
| ID0AAH7CG11ZM1 | gi|82579683|gb|DV751339.1|DV751339 | A1SU36 | | 1.00E-42 | | Hypothetical protein | |  |  |  | 1.4 |
| ID0AAK10YD02CM1 | CL1910Contig1 | Q3LB64 | | 1.00E-65 | | Hypothetical protein | |  |  | -5.8 | -9.0 |
| ID0AAK10YM15CM1 | CL1Contig691 | Q7QF07 | | 8E-18 | | Hypothetical protein | |  |  | 2.0 | 2.8 |
| ID0AAK10YM20CM1 | CL2869Contig1 | Q9VX62 | | 8E-16 | | Hypothetical protein | |  |  | 2.0 | 2.6 |
| ID0AAK1YE06CM1 | gi|86461613|gb|DY227485.1|DY227485 | A7UTQ3 | | 1.00E-29 | | Hypothetical protein | |  |  | -1.8 |  |
| ID0AAK1YG07CM1 | CL1Contig135 | Q17PZ7 | | 3E-11 | | Hypothetical protein | |  |  | -1.6 |  |
| ID0AAK1YG09CM1 | CL12348Contig1 | Q9VV23 | | 4.00E-07 | | Hypothetical protein | |  |  | -3.2 | -3.0 |
| ID0AAK4YC17CM1 | gi|86462588|gb|DY228460.1|DY228460 | A7SQS6 | | 2.00E-10 | | Hypothetical protein | |  |  |  | 5.5 |
| ID0AAK4YH22CM1 | CL7471Contig1 | A0NCC8 | | 4.00E-10 | | Hypothetical protein | |  |  | -1.5 |  |
| ID0AAK4YJ03CM1 | gi|86462723|gb|DY228595.1|DY228595 | Q5TRZ9 | | 1.00E-36 | | Hypothetical protein | |  |  | -1.8 | 1.8 |
| ID0AAK5YC06CM1 | CL1Contig731 | A7K920 | | 5.00E-09 | | Hypothetical protein | |  |  | -5.5 | -33.3 |
| ID0AAK5YG07CM1 | CL1227Contig1 | A5EIF1 | | 1E-12 | | Hypothetical protein | |  |  |  | 2.4 |
| ID0AAK5YM14CM1 | CL24Contig3 | Q16GC0 | | 4E-13 | | Hypothetical protein | |  |  | 229.9 | 59.2 |
| ID0AAK6YD24CM1 | CL1Contig582 | Q9VKQ2 | | 6.00E-23 | | Hypothetical protein | |  |  |  | 1.3 |
| ID0AAK6YE03CM1 | CL78Contig2 | Q9VN72 | | 6.00E-59 | | Hypothetical protein | |  |  |  | 1.8 |
| ID0AAK6YK16CM1 | CL5957Contig1 | Q16FI1 | | 1.00E-145 | | Hypothetical protein | |  |  |  | 1.3 |
| ID0AAK8YG11CM1 | CL3667Contig1 | Q0IF16 | | 1.00E-96 | | Hypothetical protein | |  |  |  | 1.3 |
| ID0AFF13AH05CM1 | CL641Contig1 | A8Q7J0 | | 2.00E-60 | | Hypothetical protein | |  |  | 1.3 |  |
| ID0AFF2BA02CM1 | gi|82565025|gb|DV740715.1|DV740715 | Q9VIL0 | | 5.00E-20 | | Hypothetical protein | |  |  | 1.3 |  |
| ID0AFF4AA03CM1 | CL1563Contig1 | Q7Q787 | | 2E-15 | | Hypothetical protein | |  |  | -1.3 | -1.4 |
| ID0AFF5AH06CM1 | CL1Contig152 | Q9VT05 | | 2E-11 | | Hypothetical protein | |  |  | 1.4 | 1.3 |
| ID0AFF7DB05CM1 | CL1Contig868 | Q9V3V2 | | 8.00E-74 | | Hypothetical protein | |  |  | 2.0 | 1.3 |
| ID0AFF7DG11CM1 | CL606Contig1 | Q9VW32 | | 1.00E-120 | | Hypothetical protein | |  |  | 1.9 | -1.4 |
| MpW-I-C12 | CTG_MP_47.3-34597535 | B1U3M9 | | 1.00E-13 | | Hypothetical protein | |  |  | -3.1 |  |
| MpW-II-H12 | CL_MP_5725.3-34966026 | Q4W1G5 | | 1.00E-13 | | OS-D-like protein | |  |  | -2.0 |  |
| MpW-III-G05 | CL_MP_7110.3-34966159 | A9P2J5 | | 8.00E-12 | | Hypothetical protein | |  |  |  | -3.1 |
| MpW-VI-H09 | CTG_MP_29.3-34597939 | Q9VEZ6 | | 2.00E-33 | | Hypothetical protein | |  |  | -10.0 | -9.6 |
| ID0AAK1YG18CM1 | CL2735Contig1 | Q9VI06 | | 2.00E-22 | | Osiris 19 | |  |  | 2.6 | -2.9 |
| ApAL3SD-X-B8 | CL1Contig384 | Q9VNM7 | | 2.00E-30 | | Osiris 7 | |  |  | 2.1 | 2.0 |
| ID0AAK3YL07CM1 | CL1Contig971 | Q9VNM9 | | 5.00E-21 | | Osiris 9 | |  |  | 32.4 | 5.2 |
| ID0AFF15DB08CM1 | CL246Contig1 | Q9VPW8 | | 3.00E-20 | | Smell impaired 21F | |  |  | -1.7 | -1.8 |
| ApAL3SD-XI-E1 | CL1Contig233 | Q9VHT2 | | 9.00E-42 | | Tex | | 1.4 |  |  | 3.5 |
| ApAL3SD-XIV-C6 | CL1674Contig1 | Q7K0K1 | | 5.00E-37 | | Hypothetical protein | |  |  |  | 1.8 |
| ID0AAK1YD10CM1 | CL356Contig1 | Q1ZZR0 | | 2.00E-81 | | Mago nashi | |  |  |  | 1.4 |
| ApHL3SD-XXXIII-E10 | CL684Contig2 | Q4W449 | | 6.00E-63 | | OS-D-like | |  |  | -2.2 | -1.8 |
| MpW-XVIII-G05 | CL_MP_7376.3-34966851 | Q56FC3 | | 2.00E-10 | | Hypothetical protein | |  |  | 1.3 |  |

The name of each regulated EST, the corresponding contig or singleton name, the Uniprot accession and the *e*-value of the corresponding protein, its putative function, as well as the regulation factor are indicated for each time-course.
