# Supplementary material for: Transcriptomic and proteomic analyses of seasonal photoperiodism in the pea aphid
Source: BMC Genomics. 2009 Sep 29;10:456. doi: 10.1186/1471-2164-10-456 (PMC2763885; doi:10.1186/1471-2164-10-456)
Supplement: Additional file 3 — Type of RR domain contained in the 38 regulated transcripts encoding cuticular proteins. [file 1471-2164-10-456-S3.DOC]

**Additional File 3 - Type of RR domain contained in the 38 regulated transcripts encoding cuticular proteins**.

| **EST name** | **Contig name** | **L4-G0** | **A-G0** | **L2-G1** | **L4-G1** | **Type of domain** |
| --- | --- | --- | --- | --- | --- | --- |
|  |  |  |  |  |  |  |
|  |  |  |  |  |  |  |
| ID0AAK4YO19CM1 | CL1115Contig1 |  |  |  | -3.4 | RR1 |
| ID0AAK1YD01CM1 | CL12Contig1 |  | 2.3 | -4.1 | 2.2 | **RR1** |
| ApHL3SD-XXXI-F9 | CL1Contig281 |  | 1.4 | -3.0 | -2.8 | **RR1** |
| ID0AAK1YE23CM1 |  |  | 1.8 | -5.0 | -8.3 |  |
| ID0AAK1YB23CM1 | CL1Contig856 |  |  | -3.7 | -6.3 | **RR1** |
| ApAL3SD-II-D1 | CL67Contig1 |  |  | -2.4 | -6.1 | **RR1** |
| ID0AAK4YM19CM1 | gi|86462801|gb|DY228673.1|DY228673 |  |  | -4.5 | -9.2 | **RR1** |
| ID0AAK8YK19CM1 | gi|86464122|gb|DY229994.1|DY229994 |  |  | -6.7 | -5.5 | **RR1** |
|  |  |  |  |  |  |  |
|  |  |  |  |  |  |  |
| ApAL3SD-VI-A11 | CL17Contig2 |  |  | -1.9 | -8.3 | **RR2** |
| ID0AAK4YB22CM1 |  | -2.3 |  | -3.6 | -112.5 |  |
| ID0AAK4YG05CM1 | CL17Contig3 | -2.0 |  | -3.0 | -13.8 | **RR2** |
| ID0AAK4YH18CM1 | CL1Contig1043 | -3.9 | 3.1 | -7.8 |  | **RR2** |
| ApAL3SD-XX-F7 | CL1Contig1146 |  |  | -2.2 | -4.6 | **RR2** |
| ApHL3SD-XXVII-B5 | CL1Contig1247 | -1.8 |  | -4.7 | -14.7 | **RR2** |
| ID0AAK2YK14CM1 |  | -3.1 |  | -6.5 | -125.8 |  |
| ID0AAK3YF14CM1 | CL1Contig1346 | -2.0 |  | -4.4 | -49.2 | **RR2** |
| ID0AAK4YI23CM1 | CL1Contig279 |  |  |  | 1.8 | **RR2** |
| ApAL3SD-XVI-G10 | CL1Contig454 | -1.8 |  | -4.9 | -19.6 | **RR2** |
| ApAL3SD-III-F4 | CL1Contig476 |  |  | 1.7 | -1.9 | **RR2** |
| ApHL3SD-I-D1 | CL1Contig490 |  |  | -2.4 | -3.0 | **RR2** |
| ApAL3SD-XIX-D8 | CL1Contig631 | -1.6 |  | -2.9 | -4.6 | **RR2** |
| ID0AAK1YF06CM1 | CL1Contig690 | -3.5 |  | -9.3 | -38.4 | **RR2** |
| ID0AAK2YA19CM1 | CL1Contig707 |  |  |  | -55.0 | **RR2** |
| ID0AAK5YN01CM1 | CL1Contig858 | -3.5 |  | -9.9 | -500.3 | **RR2** |
| ID0AAK4YM05CM1 | CL1Contig990 | -2.7 |  | -7.1 |  | **RR2** |
| ApAL3SD-XII-A2 | CL568Contig1 |  |  | 1.5 |  | **RR2** |
| ApHL3SD-XXVIII-D1 | CL667Contig1 | -1.5 |  | -1.5 | -6.0 | **RR2** |
| ID0AFF12BC01CM1 |  |  |  | -1.7 | -6.8 |  |
| ApAL3SD-XVIII-B9 | CL778Contig1 |  |  |  | -2.6 | **RR2** |
|  |  |  |  |  |  |  |

Each regulated cDNA is named by its EST accession number and its corresponding contig name. The type of domain (RR1, RR2 or no domain) is indicated for each transcript as well as its level of regulation at the 4 developmental stages of the kinetics.
