# Supplementary material for: Transcriptomic and proteomic analyses of seasonal photoperiodism in the pea aphid
Source: BMC Genomics. 2009 Sep 29;10:456. doi: 10.1186/1471-2164-10-456 (PMC2763885; doi:10.1186/1471-2164-10-456)
Supplement: Additional file 4 — List of significant GO terms. [file 1471-2164-10-456-S4.DOC]

**Additional File 4 - list of significant GO terms.**

|  | **Term** | **#1 vs #2** | **p value** | **Adjusted p value** |
| --- | --- | --- | --- | --- |
| *GO biological process at level 3* | |  |  |  |
|  | biosynthetic process (GO:0009058) | 32.37% 67.63% | 4.91969e-09 | 2.75503e-07 |
|  | macromolecule metabolic process (GO:0043170) | 42.39% 57.61% | 4.26073e-05 | 0.001193 |
|  | establishment of localization (GO:0051234) | 66.81% 33.19% | 0.00104027 | 0.0194184 |
|  | cell communication (GO:0007154) | 75.34% 24.66% | 0.0014732 | 0.0206248 |
|  | primary metabolic process (GO:0044238) | 45.15% 54.85% | 0.00207153 | 0.0232011 |
|  | cellular metabolic process (GO:0044237) | 45.89% 54.11% | 0.00377244 | 0.0352094 |
|  | regulation of biological process (GO:0050789) | 65.62% 34.38% | 0.00445302 | 0.0356242 |
| *GO biological process at level 4* | |  |  |  |
|  | cellular biosynthetic process (GO:0044249) | 31.67% 68.33% | 1.44683e-09 | 1.75067e-07 |
|  | cellular macromolecule metabolic process (GO:0044260) | 37.08% 62.92% | 3.67385e-08 | 2.22268e-06 |
|  | protein metabolic process (GO:0019538) | 38.35% 61.65% | 1.00098e-06 | 4.03727e-05 |
|  | transport (GO:0006810) | 66.52% 33.48% | 0.00140242 | 0.0424231 |
| *GO cellular component at level 3* | |  |  |  |
|  | non-membrane-bound organelle (GO:0043228) | 33.15% 66.85% | 1.88009e-08 | 2.06809e-07 |
|  | membrane-bound organelle (GO:0043227) | 62.17% 37.83% | 0.000784068 | 0.00431237 |
| *GO molecular function at level 3* | |  |  |  |
|  | structural constituent of ribosome (GO:0003735) | 19.14% 80.86% | 2.57667e-15 | 1.59753e-13 |
|  | structural constituent of cuticle (GO:0042302) | 12.47% 87.53% | 2.18062e-07 | 6.75991e-06 |

GO terms were compared between all the spotted cDNAs and the significantly regulated cDNAs having a homolog in *D. melanogaster*. Only levels 3 and/or 4 are shown. Adjusted p value < 0.05). #1: spotted cDNA set; “2: significantly regulated cDNA set.
